# Disruption of dopamine transmission by cholesterol depletion is associated with alterations in protein lipid raft partitioning and actin dynamics

**DOI:** 10.1016/j.neuropharm.2026.111078

**Published:** 2026-06-13

**Authors:** Anna I. Neel, Evelyn T. Lee, Alyssa M. West, Monica H. Dawes, Rebekah D. Schlitzer, Reid A. Suddaby, Glen S. Marrs, Sara R. Jones, Rong Chen

**Affiliations:** a Department of Translational Neuroscience, Wake Forest University School of Medicine, Winston Salem, NC, 27157, USA; b Department of Biology, Wake Forest University, Winston Salem, NC, 27109, USA

**Keywords:** Cholesterol, Dopamine transporter, Dopamine reuptake, Dopamine release, Lipid rafts, Actin polymerization

## Abstract

Disrupted brain cholesterol homeostasis is implicated in neurological disorders involving aberrant dopamine (DA) signaling; however, the direct effects of cholesterol on DA transmission in native tissue have not yet been demonstrated. Using *ex vivo* fast-scan cyclic voltammetry in nucleus accumbens slices from male rats, we found that membrane cholesterol depletion with methyl-β-cyclodextrin (MβCD,3–10 mM) significantly reduced evoked DA release and decreased the apparent maximal rate of DA reuptake via the dopamine transporter (DAT). Because cholesterol is critical for the formation of lipid raft microdomains, cholesterol depletion could disrupt DA transmission by altering the membrane localization of proteins involved in neurotransmitter release. Using sucrose density gradient fractionation, we found that MβCD decreased the raft association of vesicle-associated membrane protein 2 (VAMP2) without altering the localization of syntaxin-1A, synaptosomal-associated protein 25, synaptotagmin-1, and N-type voltage-gated calcium channels. Therefore, MβCD may reduce DA release by disrupting localization of VAMP2, a core component of the vesicle fusion machinery. We also examined actin polymerization, a key regulator of vesicle docking and fusion, and found that MβCD treatment decreased actin polymerization, as evidenced by an increased globular-to-filamentous actin ratio and reduced phalloidin labeling of filamentous actin in striatal slices. Finally, although DAT lipid raft localization was unchanged, MβCD attenuated cocaine’s ability to inhibit DAT reuptake function, suggesting that cholesterol depletion disrupts the outward-facing conformation of DAT required for high-affinity ligand binding. Overall, these findings provide new mechanistic insights into how cholesterol depletion may contribute to dysregulated DA signaling in diseases involving altered brain cholesterol metabolism.

## 1. Introduction

Emerging evidence has implicated altered brain cholesterol metabolism in several neurological disorders involving disrupted dopamine (DA) signaling, including Parkinson’s disease, Huntington’s disease, schizophrenia, and substance use disorder (Alsebaaly et al., 2018; Block et al., 2010; Huang et al., 2019; Inabinett et al., 2025; Kreilaus et al., 2016). Cholesterol is a major structural component of neuronal membranes and is an important regulator of neurotransmission. Although the brain accounts for only 2% of total body mass, it contains nearly 23% of the body’s cholesterol content (Dietschy, 2009). Because cholesterol does not cross the blood-brain barrier, the brain relies on tightly regulated *de novo* synthesis to maintain cholesterol homeostasis (Vitali et al., 2014). In the brain, cholesterol is primarily, if not exclusively, present in its free, unesterified form (Björkhem and Meaney, 2004), in contrast to peripheral tissue, where cholesterol is mainly stored and transported as cholesteryl esters. Approximately 80% of brain cholesterol is found in myelin sheaths, with a slow turnover rate of approximately 0.4% per day (Dietschy and Turley, 2001). The remaining cholesterol is localized within cell membranes, where turnover is nearly 30% per day, suggesting that membrane cholesterol is dynamic and subject to regulation (Björkhem and Meaney, 2004; Dietschy, 2009; Dietschy and Turley, 2001). Notably, cholesterol is not evenly distributed on the cell membrane. This heterogeneity gives rise to specialized membrane regions with distinct structural and functional properties, including lipid raft and non-raft microdomains. Lipid raft microdomains are enriched in cholesterol and sphingolipids, which allow for the assembly and organization of membrane proteins and signaling molecules that facilitate efficient signal transduction (Sapoń et al., 2023; Warda et al., 2025). Specifically, lipid raft microdomains promote the assembly of proteins involved in vesicle fusion, neurotransmitter release, and membrane transport (Korade and Kenworthy, 2008; Lang et al., 2001; Orth and Bellosta, 2012). Given its critical role at the membrane, disruption of cholesterol homeostasis could therefore alter the presynaptic mechanisms that regulate DA signaling.

DA transmission depends on two tightly coordinated processes: vesicular DA release and dopamine transporter (DAT)-mediated DA reuptake. DA release is triggered by presynaptic calcium (Ca^2+^) influx, primarily through N-type channels such as CaV2.2 (Bergquist et al., 1998; Phillips and Stamford, 2000; Robinson et al., 2010). Ca^2+^ binding to synaptotagmin-1 triggers assembly of the canonical SNARE complex, consisting of the v-SNARE vesicle-associated membrane protein 2 (VAMP2) on synaptic vesicles and the t-SNAREs syntaxin-1A and synaptosomal-associated protein 25 (SNAP-25) on the plasma membrane (Banerjee et al., 2020). Formation of this complex drives vesicle fusion and exocytosis (Lv et al., 2008; Rowe et al., 2001). After release, extracellular DA is cleared by DAT for repackaging or degradation. Because both release and DAT-mediated reuptake occur within the terminal of DA neurons, each process is likely to be sensitive to alterations in membrane cholesterol.

Several lines of evidence support a role for cholesterol in regulating both DA release and DAT-mediated reuptake. For instance, in PC12 cells, acute depletion of membrane cholesterol using methyl-β-cyclodextrin (MβCD) reduces potassium chloride- and ATP-evoked DA release (Chamberlain et al., 2001; Lang et al., 2001). To date, few, if any, studies have examined the effects of cholesterol depletion on evoked DA release in animal brain tissue. Cholesterol also influences DA reuptake through its effects on DAT. Structural and computational studies indicate that the presence of cholesterol promotes the outward-facing conformation of DAT, which favors high-affinity substrate binding and the binding of inhibitors such as cocaine (Penmatsa et al., 2013; Zeppelin et al., 2018). Consistent with this, cholesterol depletion using MβCD decreases DAT-mediated DA reuptake and attenuates cocaine inhibition of DAT in HEK293 cells, N2A cells, and rat striatal synaptosomes (Hong and Amara, 2010; Jones et al., 2012; Wang et al., 2023). In striatal slices, indirectly altering cholesterol availability with probucol, which inhibits cholesterol efflux, also impairs DAT-dependent DA reuptake (Threlfell et al., 2021). Collectively, these studies show that cholesterol contributes to both DA release and reuptake; however, whether direct cholesterol depletion disrupts both of these coordinated processes in native striatal tissue has yet to be explored. Because DA signaling arises from the dynamic balance between release and reuptake, measuring both processes simultaneously is essential for determining how cholesterol depletion alters overall DA dynamics (Sulzer et al., 2016). Such insights may provide a mechanistic foundation for understanding neurological disorders associated with disrupted cholesterol homeostasis.

One mechanism by which cholesterol depletion may impair DA transmission is by disrupting the membrane organization of proteins involved in DA release and reuptake. Namely, lipid rafts serve as platforms for assembling the proteins that facilitate vesicle fusion and exocytosis, and cholesterol depletion via MβCD alters the raft association of several proteins involved in this process (Chamberlain et al., 2001; Gil et al., 2005; Lang, 2007). For example, treatment with saponin, which sequesters cholesterol, fully displaced syntaxin-1A, SNAP-25, and VAMP2 from lipid rafts in PC12 cells (Chamberlain et al., 2001). Further, high concentrations of MβCD removed 90% of membrane cholesterol and partially displaced these same SNARE proteins, along with synaptotagmin-1, from raft fractions in rat synaptosomes (Gil et al., 2005). Moreover, DAT is distributed across both lipid raft and non-raft microdomains; however, the effects of MβCD on DAT raft association are not consistently observed across cell types, including LLC-PK_1_, HEK293, and MN9D cells (Foster et al., 2008; Hong and Amara, 2010; Jones et al., 2012). Importantly, the effects of cholesterol depletion on DAT raft association in animal brain tissue have yet to be investigated. Therefore, the goal of the present study was to determine whether cholesterol depletion would alter both DA release and reuptake in striatal tissue and identify the molecular components involved in these processes.

Cholesterol may also regulate DA terminal function through effects on the actin cytoskeleton. Actin exists in a dynamic equilibrium between globular (G)-actin monomers and filamentous (F)-actin polymers (Li et al., 2024; Mundhara et al., 2019). The continuous cycling between these two states is critical for multiple aspects of vesicular release, including vesicle mobilization from reserve pools, trafficking to active zones, and positioning of vesicles for docking and fusion (Dillon and Goda, 2005). At presynaptic terminals, F-actin provides cytoskeletal “tracks” that guide the movement of synaptic vesicles to the readily-releasable pool (Prekeris and Terrian, 1997; Zhang and Augustine, 2021). Disruption of this G/F actin balance can therefore impair efficient vesicle organization and release (Rust and Maritzen, 2015). Membrane cholesterol appears to be important for the regulation of actin dynamics, as MβCD has been shown to decrease actin polymerization in MN9D cells, HeLa cells, and hippocampal cultures (Hering et al., 2003; Li et al., 2015; Mundhara et al., 2019). In turn, actin contributes to the organization of lipid rafts: for example, pharmacological disruption of F-actin polymerization has been shown to reduce the raft association of CaV2.2 in COS-7 cells (Robinson et al., 2010). These observations suggest that cholesterol depletion could impair DA transmission in part by disrupting actin-dependent membrane organization.

In the present study, we used *ex vivo* fast-scan cyclic voltammetry in rat nucleus accumbens slices together with biochemical assays in striatal synaptosomes to test whether acute cholesterol depletion disrupted evoked DA release and reuptake and whether these functional effects were accompanied by changes in lipid raft organization and actin polymerization. To our knowledge, this is the first study to concurrently assess changes in both DA release and reuptake following cholesterol depletion in native tissue while also examining the underlying mechanisms. Because overall DA signaling is determined by the dynamic interplay between these two tightly coordinated processes, our findings may provide a more holistic understanding of how cholesterol regulates striatal DA transmission.

## 2. Materials and methods

### 2.1. Animals

Male Sprague-Dawley rats (250–350 g; Envigo, Indianapolis, IN, USA) were housed under temperature- and humidity-controlled conditions on a reverse 12:12 h light/dark cycle (lights on at 0400 h; lights off at 1600 h). Food and water were available *ad libitum*. All procedures were conducted in Association for Assessment and Accreditation of Laboratory Animal Care-accredited facilities in accordance with the National Institutes of Health *Guide for the Care and Use of Laboratory Animals*. All experimental protocols were approved by the Institutional Animal Care and Use Committee at Wake Forest University School of Medicine.

### 2.2. Ex vivo fast-scan cyclic voltammetry

*Ex vivo* fast-scan cyclic voltammetry (FSCV) was used to assess electrically evoked DA release and reuptake in rat brain slices containing the nucleus accumbens (NAc) core, as described previously (Mauterer et al., 2018). All animals were euthanized 4 h into the light phase, and brains were rapidly removed and placed in oxygenated artificial cerebrospinal fluid (aCSF) containing 126 mM NaCl, 2.5 mM KCl, 1.2 mM NaH_2_PO_4_·H_2_O, 2.4 mM CaCl_2_·2H_2_O, 1.2 mM MgCl_2_·6H_2_O, 25 mM NaHCO_3_, 0.4 mM ascorbic acid, and 11 mM D-glucose. Coronal brain slices (400 μm) containing the NAc core were prepared using a vibrating tissue slicer (Leica VT1200S, Leica Biosystems, Wetzlar, Germany) and transferred to a recording chamber superfused with oxygenated aCSF (32°C, 1 mL/min). A carbon-fiber microelectrode (100–200 μm in length, 7 μm in diameter) and a bipolar stimulating electrode (MS303–3-B-SPC, Plastics One, Roanoke, VA, USA) were positioned in the NAc core. Endogenous DA release was evoked every 3 min using a single electrical pulse (750 μA, 4 ms). This stimulation intensity was selected to produce strong evoked DA signals across recording sites in the NAc core while maintaining stable repeated measurements throughout the drug treatment and avoiding floor effects during treatments expected to reduce DA release. This intensity is consistent with prior *ex vivo* FSCV studies from our group using peri-maximal single-pulse stimulation at approximately 700 μA (Dawes et al., 2024; Kuiper et al., 2024). The DA signal was detected by applying a triangular voltage waveform (−0.4 to +1.2 to −0.4 V vs Ag/AgCl) at 400 V/s to the recording electrode.

Once evoked DA signals stabilized across three consecutive recordings (defined as <20% variation across >3 consecutive collections), baseline DA release and reuptake were determined. Slices were then perfused for 1 h with oxygenated aCSF containing MβCD (0.3, 3, or 10 mM; C4555, Millipore-Sigma, St. Louis, MO, USA), followed by washout with aCSF until responses again stabilized. Concentrations and incubation times of MβCD were selected based on previous literature using *ex vivo* slice preparations (Assaife-Lopes et al., 2014; Ramadhanti et al., 2021; Wang and Schreurs, 2010). After responses re-stabilized, cocaine hydrochloride (0.3–30 μM; National Institute on Drug Abuse, Bethesda, MD, USA) was cumulatively applied to slices for 18 collections (~54 min) per concentration to assess inhibition of DA reuptake as previously described (Estave et al., 2024; Siciliano et al., 2019). Evoked DA signals were modeled using Michaelis-Menten kinetics to estimate the apparent maximal rate of reuptake (V_*max*_) and apparent *K*_*m*_. Recording electrodes were calibrated using a flow-injection system with 3 μM DA. Data analysis was performed using Demon Voltammetry and Analysis software (Yorgason et al., 2011).

Baseline FSCV experiments included 1–2 slices per rat from 8 to 12 animals per group. A subset of these animals was used for cocaine inhibition experiments (*n* = 6–8 rats per group, 1–2 slices per rat). For all FSCV analyses, individual slices were treated as independent data points because local DA release and reuptake parameters vary across recording sites within the NAc core to an extent comparable to across-animal variability (Calipari et al., 2012; Dawes et al., 2024).

### 2.3. Striatal synaptosome preparation

To examine the mechanisms underlying the DA terminal deficits observed with FSCV, we applied the same MβCD concentrations used in slice experiments to striatal synaptosomes. This preparation allowed for more direct biochemical assessment of mechanisms while minimizing the variability associated with tissue thickness and drug penetration in intact slices (Jhou and Tai, 2017; Voss et al., 2013). Additionally, the FSCV paradigm employs repeated electrical stimulation over several hours, which may confound downstream biochemical assessment of membrane protein organization.

Brains were rapidly removed and placed on a cold rat brain matrix, and 1 mm thick coronal slices were obtained. The NAc and dorsal striatum were dissected using anatomical landmarks. These two regions were pooled from each animal to generate enough crude synaptosome for detection of protein compartmentalization in lipid rafts using sucrose density gradient fractionation assays.

Striatal tissue was placed in ice-cold homogenization buffer containing 0.32 M sucrose and 4 mM HEPES (pH 7.4) supplemented with protease inhibitors (P8465; Millipore-Sigma, Burlington, MA, USA) as previously described (Bermejo et al., 2014). Homogenates were centrifuged at 800 × g for 10 min at 4°C to remove nuclei and large debris. The resulting supernatant was centrifuged at 12,000 × g for 15 min at 4°C to obtain crude synaptosome pellets. Pellets were resuspended in oxygenated Krebs-Ringer buffer (KRB), containing 145 mM NaCl, 2.7 mM KCl, 1.2 mM KH_2_PO_4_, 24.9 mM NaHCO_3_, 1.0 mM MgCl_2_, 1.2 mM CaCl_2_·2H_2_O, and 10 mM glucose, pH 7.4, supplemented with protease inhibitors.

### 2.4. Synaptosome treatment with methyl-β-cyclodextrin

Samples were treated with MβCD (0.3, 3, or 10 mM) prepared in KRB containing protease inhibitors and incubated at 37°C for 30 min with gentle agitation. The concentrations and incubation times for MβCD were selected based on prior studies using striatal membrane preparations (Hong and Amara, 2010; Morissette et al., 2018; Teixeira et al., 2012). Incubation time was shortened to 30 min compared to 1 h for FSCV experiments to account for the increased membrane accessibility for drugs in synaptosomes (Borisova, 2013; Tarasenko et al., 2010). After incubation, reactions were terminated by adding ice-cold KRB, and samples were centrifuged at 12,000 x g for 15 min at 4°C. Supernatants were discarded, and pellets were resuspended in the appropriate buffer for biochemical assays.

### 2.5. Laurdan generalized polarization assay

To determine whether cholesterol depletion with MβCD altered membrane order as defined by membrane fluidity and rigidity, we performed the Laurdan generalized polarization (GP) assay in synaptosomes as previously described (Vošahlíková et al., 2021). Synaptosomes were treated with MβCD (0.3, 3, and 10 mM) to match those used in FSCV experiments, and 50 mM MβCD was included as a positive control to confirm that the Laurdan GP assay could detect changes in membrane order following substantial cholesterol depletion. After MβCD treatment, pellets were resuspended in 50 mM Tris-HCl (pH 7.6). Samples were incubated with 10 μM Laurdan (7275, Tocris Bioscience, Bristol, UK) prepared in N,N-Dimethylformamide (DMF; final DMF concentration <0.5%) for 60 min at 37°C in the dark with gentle agitation. Samples were then centrifuged, and pellets were resuspended in 50 mM Tris-HCl (pH 7.6) for the assay. Blank wells containing buffer and Laurdan only were included to control for background fluorescence.

Fluorescence emission was recorded at 440 nm and 490 nm following excitation at 350 nm. Background fluorescence from blank wells was subtracted from all measurements. Laurdan generalized polarization (GP) was calculated using:

$$GP=\frac{I_{440}-I_{490}}{I_{440}+I_{490}}$$

where I_440_ and I_490_ represent fluorescence intensities at 440 nm and 490 nm, respectively. Higher GP values correspond to increased membrane lipid order and rigidity, whereas lower GP values indicate reduced lipid order and increased membrane fluidity. GP values were averaged across triplicates and expressed as mean ± SEM.

### 2.6. Sucrose density gradient ultracentrifugation

Following MβCD treatment, synaptosome pellets were suspended in MBS-T buffer (25 mM MES, 150 mM NaCl, 1% Triton X-100, pH 6.5) containing protease inhibitors. Samples were homogenized using a Dounce homogenizer and sheared through a 25-gauge needle. Homogenates were incubated on ice for 30 min and then centrifuged at 1000 × g for 10 min at 4°C to remove insoluble material. Aliquots of the resulting supernatant were reserved for cholesterol quantification by the Amplex Red assay, while the remaining lysate was used for sucrose density gradient ultracentrifugation.

Sucrose density gradient ultracentrifugation was used to isolate raft-enriched and non-raft membrane fractions, adapted from previously established protocols (Chen et al., 2007; Gil et al., 2005). Lysates were mixed with an equal volume of 80% sucrose solution resulting in 40% sucrose in MBS buffer (1.6 mL total volume). The sample was then overlaid with 1.8 mL of 30% sucrose, followed by 800 μL of 5% sucrose. Gradients (4.2 mL final volume) were centrifuged at 200,000 × g for 18 h at 4°C using an SW55 Ti rotor (342196; Beckman Coulter, Brea, CA, USA) in an Optima XE-90 ultracentrifuge (A94471; Beckman Coulter, Brea, CA, USA). Following centrifugation, 15 equal-volume fractions were collected from the top of the gradient. Detergent-resistant, raft-enriched fractions consistently localized to the 5–30% sucrose interface (typically fractions 4–6). All fractions were stored at −80°C until further analysis.

### 2.7. Western blot

Western blotting was performed to detect the abundance of proteins in each fraction. Because non-raft fractions contain substantially more protein than raft fractions (Gil et al., 2005; Levitt et al., 2009), loading equal volumes produced disproportionately stronger signal in non-raft fractions and made concurrent quantification in both raft and non-raft fractions difficult (Supplemental Fig. 1). Therefore, loading volumes were adjusted across the gradient as follows: 35 μL for raft fractions 4–6 (flotillin-positive), 17.5 μL for fractions 7–12, and 11.67 μL for non-raft fractions 13–15 (transferrin-positive). Proteins were separated by SDS-PAGE and transferred to nitrocellulose membranes. Membranes were blocked for 1 h at room temperature in 5% non-fat milk prepared in Tris-buffered saline containing 1% Tween-20 (TBST), followed by overnight incubation at 4°C with the appropriate primary antibodies (Table 1). The next day, membranes were incubated for 1 h at room temperature with an appropriate HRP-conjugated secondary antibody. In some experiments, membranes were cut into strips to allow simultaneous probing of proteins with different molecular weights. Chemiluminescent signal was detected using Pierce ECL Western Blotting Substrate (32106; Thermo Fisher Scientific, Waltham, MA, USA) and imaged with a Bio-Rad ChemiDoc Imaging System (Bio-Rad Laboratories, Hercules, CA, USA). Band intensities were quantified using ImageJ (National Institutes of Health, Bethesda, MD, USA), and data were expressed as the percentage of total signal present in flotillin-positive fractions (fractions 4–6) relative to the total signal across all fractions and normalized to control. Flotillin was confirmed to be present only in fractions 4–6 (Supplemental Fig. 2).

For total protein expression, 30 μg of protein per sample was loaded onto SDS-PAGE gels, and western blotting was performed as described above. Band intensities were quantified using ImageJ and normalized to GAPDH to account for loading variability. Data are expressed relative to control and presented as mean ± SEM.

### 2.8. Amplex Red Cholesterol assay

Aliquots of synaptosomal lysate were used to quantify free (unesterified) cholesterol, as 99.5% of brain cholesterol exists in its unesterified form (Björkhem and Meaney, 2004). Protein concentrations were measured using the Pierce BCA Protein Assay Kit (23225; Thermo Fisher Scientific, Waltham, MA). Free cholesterol content was quantified using the Amplex Red Cholesterol Assay Kit (A12216; Invitrogen, Carlsbad, CA) according to the manufacturer’s instructions. Briefly, samples were diluted in the kit’s reaction buffer and incubated with Amplex Red working solution (150 μM Amplex Red, 1 U/mL cholesterol oxidase, and 1 U/mL horseradish peroxidase) at 37°C for 30 min. This reaction produces resorufin, a fluorescent product, which was measured using a SpectraMax i3x plate reader (Molecular Devices, San Jose, CA, USA) with excitation at 560 nm and emission at 590 nm. Free cholesterol concentrations were calculated from a standard curve and normalized to sample protein content. Cholesterol concentrations were averaged across triplicates and expressed as mean ± SEM.

### 2.9. G:F-actin centrifugation assay in striatal synaptosomes

To determine the effect of MβCD treatment on actin polymerization, a G:F-actin sedimentation assay was performed to fractionate globular and polymerized actin according to the manufacturer’s instructions (BK037; Cytoskeleton Inc., Denver, CO, USA). Lysis and F-actin stabilization buffer (LAS1) was prepared using 50 mM PIPES (pH 6.9), 50 mM KCl, 5 mM MgCl_2_, 5 mM EGTA, 5% glycerol, 0.1% Nonidet P-40, 0.1% Triton X-100, 0.1% Tween-20, and 0.1% 2-mercaptoethanol, adjusted to pH 6.9. The working buffer (LAS2) was prepared by supplementing LAS1 with 1 mM ATP and protease inhibitors.

Striatal synaptosomes were treated with 3 mM MβCD, followed by centrifugation and resuspension of pellets in LAS2 at a 1:10 (w/v) pellet: buffer ratio. Homogenates were incubated in LAS2 at 37°C for 10 min to stabilize polymerized actin and centrifuged at 350 × g for 5 min to pellet tissue debris. The resulting supernatant was then ultracentrifuged at 100,000 × g for 1 h at 37°C, yielding a supernatant containing detergent-soluble G-actin and a pellet containing detergent-insoluble F-actin. The F-actin pellet was resuspended in an equal volume of freshly prepared 8 M urea and incubated at 4°C for 1 h with gentle rotation to depolymerize F-actin into G-actin prior to western blotting. Equal volumes of G- and F-actin fractions were mixed with 5x SDS sample buffer and heated at 55°C for 5 min. For immunoblotting, 10 μL of each fraction was resolved by SDS-PAGE and transferred to membranes. The G:F-actin ratio was calculated for each sample, and data are expressed relative to vehicle control as mean ± SEM.

### 2.10. Phalloidin labeling of F-actin in striatal slices

Rat brains were rapidly removed after anesthesia, embedded in optimal cutting temperature compound, and stored at −80°C until sectioning. Coronal sections (12 μm) containing the NAc were equilibrated in aCSF for 10 min, then treated with 3 mM MβCD or vehicle at 37°C for 60 min. Sections were washed, fixed in 4% paraformaldehyde for 1.5 h on ice, and then permeabilized and blocked for 1.5 h in 10% normal goat serum with 0.3% Triton X-100. F-actin was labeled with Alexa Fluor 488-phalloidin (A12379; Invitrogen, Carlsbad, CA, USA) for 2 h at room temperature in the dark, and slides were mounted with ProLong^™^ Gold Antifade containing DAPI (P36931, Thermo Fisher Scientific, Waltham, MA, USA).

Imaging was performed using a Zeiss LSM 710 confocal microscope equipped with a 20x (0.8 NA) objective. Confocal z-stacks encompassing the 12 μm slice tissue depth were acquired using identical laser power, detector gain, and pinhole settings (1.4 AU) for DAPI and Alexa Fluor 488 across all groups. Z-stacks were converted into maximum intensity projections for image analysis. Each channel was subjected to a uniform thresholding value to distinguish signal from background for quantification. For each section, three equal-area regions of interest (ROIs) were selected within the NAc. Phalloidin fluorescence intensity was normalized to DAPI signal. ROI intensity values were averaged for each section (n = 4 sections/group). Data are presented as relative to control.

### 2.11. Data analysis

Primary statistical analyses were performed using GraphPad Prism 10 (GraphPad Software, La Jolla, CA, USA). Data are presented as mean ± SEM. Normality was assessed using the Shapiro-Wilk test when appropriate, and all datasets met assumptions of normality. Unpaired Student’s t-tests were used to compare two groups. A one-way or two-way ANOVA was used to examine differences among groups when appropriate, followed by Tukey’s multiple-comparisons test. For cocaine concentration-response experiments, apparent K_m_ values across concentrations were analyzed using a mixed-effects model with Geisser-Greenhouse correction. Measurements were averaged for experiments that included replicates. A p value of ≤0.05 was considered statistically significant. To estimate effect sizes, Cohen’s d was used for two-group comparisons. Post hoc sensitivity analyses were performed using G*Power version 3.1.9.7 (Heinrich Heine University Düsseldorf, Düsseldorf, Germany) to determine the minimum detectable effect size for the sample sizes used in Figs. 2 and 3. These analyses assumed two-tailed independent samples t-tests with α = 0.05, power = 0.80, and equal group sizes.

## 3. Results

### 3.1. MβCD reduced evoked DA release, reuptake, and cocaine inhibition of reuptake

**Fig. 1.**
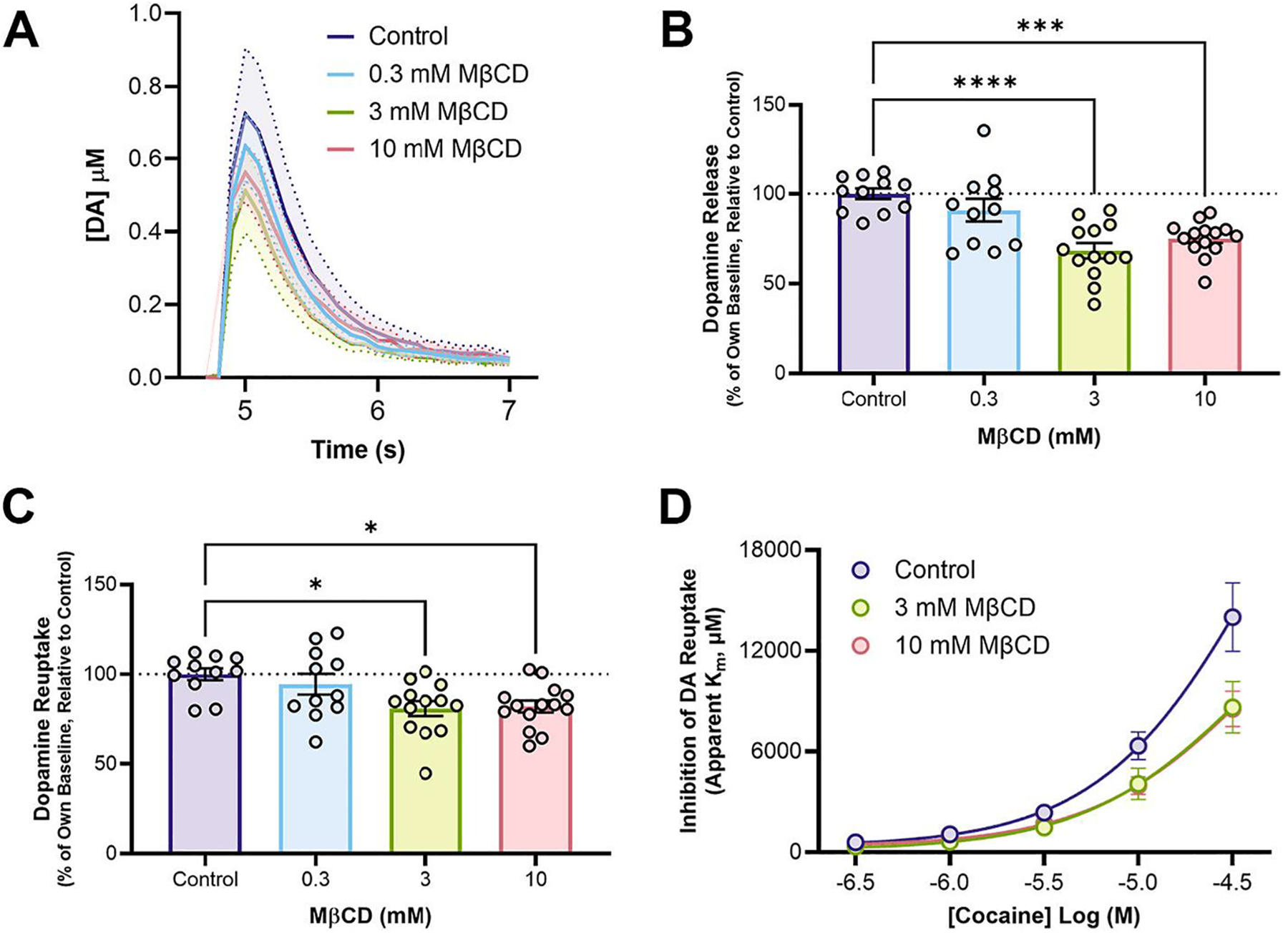
MβCD reduced evoked DA release, reuptake, and cocaine inhibition of reuptake in rat NAc slices. (A) Averaged FSCV traces illustrating treatment-dependent changes in evoked DA release and reuptake. Shading represents SEM. After treatment with control (N = 8), 0.3 (N = 8), 3 (N = 9), or 10 (N = 12) mM MβCD, evoked DA release (B) and apparent maximal reuptake rates (V_*max*_) (C) were measured. Data are expressed as percent of each slice’s own baseline, relative to control. (D) Cocaine concentration-response curves (0.3–30 μM) showing that 3 (N = 7) and 10 mM (N = 8) MβCD reduced cocaine inhibition of reuptake relative to control (N = 6). * *p* ≤ 0.05, ** *p* ≤ 0.01, *** *p* ≤ 0.001, **** *p* ≤ 0.0001. N = number of animals; 1–2 slices/animal. Data are presented as mean ± SEM.

We first tested the effect of acute cholesterol depletion with MβCD on evoked DA transmission using FSCV on slices containing the NAc core. After treatment with vehicle or MβCD (0.3, 3, 10 mM) and restabilization of the signal, evoked DA release and reuptake were measured by FSCV. Post-treatment signals were normalized to each slice’s pre-treatment signal and expressed as percent of each slice’s baseline. Averaged FSCV traces for each group are shown (Fig. 1A). A one-way ANOVA revealed a significant main effect of treatment on DA release, F_(3,45)_ = 11.94, p < 0.0001 (Fig. 1B). Tukey’s multiple comparisons test indicated that MβCD significantly reduced evoked release at 3 mM (p < 0.0001) and 10 mM (p = 0.0006) compared with control, corresponding to decreases of 31.78% and 24.90%, respectively. In contrast, 0.3 mM MβCD had no effect (p = 0.462). The 3 mM and 10 mM groups did not differ from each other (p = 0.612).

We next assessed whether MβCD affected the maximal DA reuptake rate (V_*max*_), expressed as percent of each slice’s own baseline (Fig. 1C). A one-way ANOVA revealed a significant main effect of treatment on V_*max*_, F_(3,45)_ = 4.98, p = 0.0046. Tukey’s multiple comparisons test indicated that MβCD significantly reduced V_*max*_ at 3 mM (p = 0.0134) and 10 mM (p = 0.0193) relative to control, corresponding to decreases of 19.10% and 17.98%, respectively. In contrast, 0.3 mM MβCD did not differ from the control, p = 0.8172. The 3 mM and 10 mM groups did not differ from each other, p = 0.9972.

Previous studies have demonstrated that cholesterol is necessary for maintaining the outward-facing conformation of DAT (Hong and Amara, 2010; Penmatsa et al., 2013; Wang et al., 2023; Zeppelin et al., 2018). Extracellular DA binds initially to DAT when it is in the outward-facing conformation, and cocaine preferentially binds to and stabilizes this outward-facing state. Therefore, we used cocaine as a probe to assess whether MβCD altered the availability of the outward-facing conformation of DAT in striatal tissue. To test this, we generated cocaine concentration-response curves (0.3–30 μM) and calculated apparent K_m_ values following treatment with 3 and 10 mM MβCD (Fig. 1D). A mixed-effects model with Geisser-Greenhouse correction showed significant main effects of cocaine concentration, F_(1.216, 25.85)_ = 107.2, p < 0.0001, MβCD treatment, F_(2, 23)_ = 4.522, p = 0.0221, and a cocaine x MβCD interaction, F_(8, 85)_ = 3.216, p = 0.0031. Overall, these findings demonstrated that MβCD impaired DA release and reuptake and altered the ability of cocaine to inhibit DA reuptake.

### 3.2. MβCD selectively altered the lipid raft association of proteins involved in neurotransmitter release in striatal synaptosomes

**Fig. 2.**
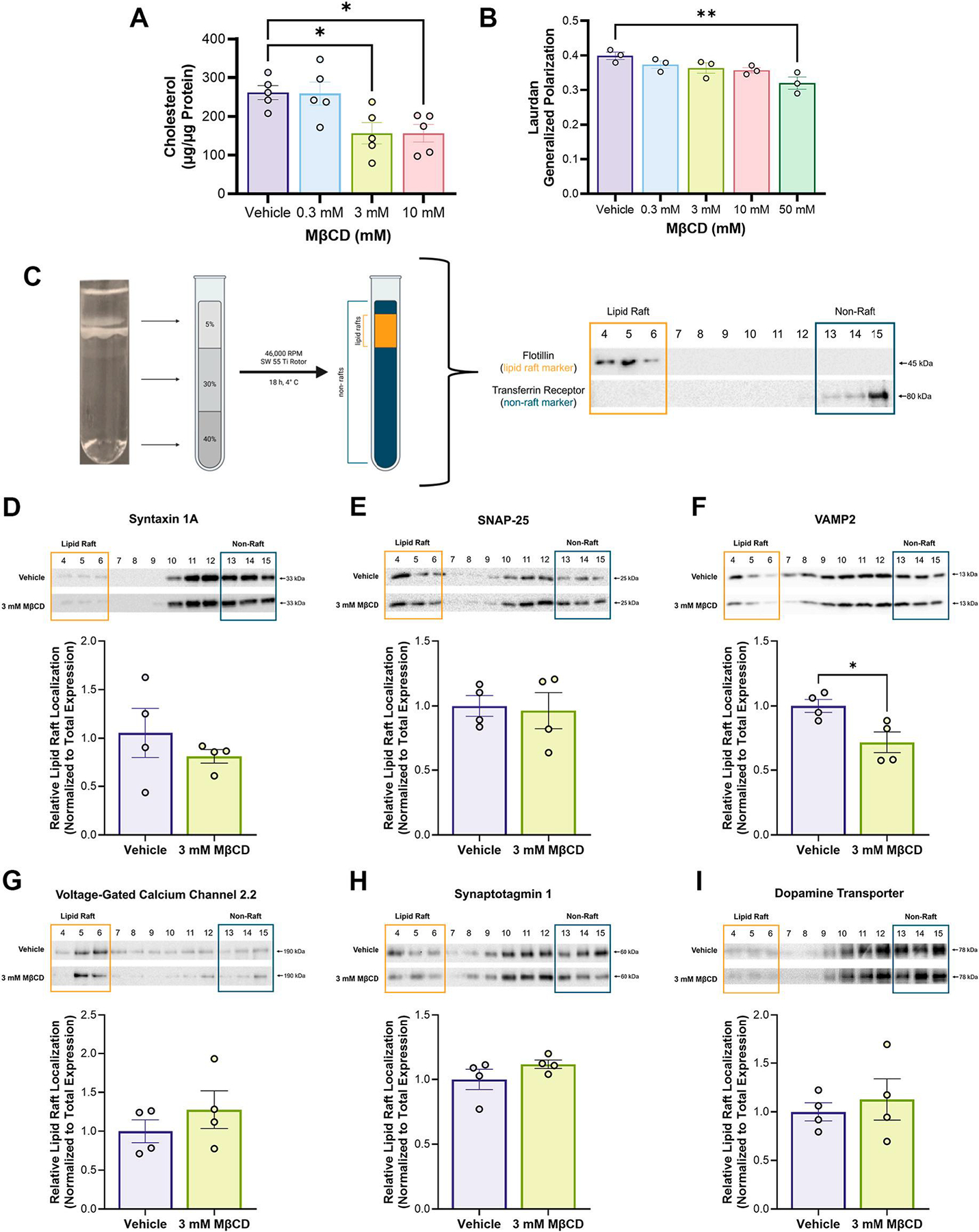
Cholesterol depletion altered the lipid raft association of proteins involved in exocytosis in striatal synaptosomes. (A) Free cholesterol measured by Amplex Red was significantly reduced at 3 and 10 mM but not at 0.3 mM. N = 5/group. (B) Membrane order measured by Laurdan GP was not altered by MβCD (0.3–10 mM), except at 50 mM. N = 3/group. (C) Schematic of sucrose density gradient ultracentrifugation workflow and representative images using flotillin to define raft fractions and the transferrin receptor to define non-raft fractions. (D-J) Raft association for each protein following treatment with 3 mM MβCD or control. Raft association was calculated as the ratio of protein signals detected in flotillin-positive raft fractions 4–6 relative to total signals across all analyzed fractions, then normalized to vehicle control. MβCD reduced the association of VAMP2 with rafts. Syntaxin-1A, SNAP-25, synaptotagmin-1, CaV2.2, and DAT showed no significant redistribution. N = 4/group. *p ≤ 0.05, **p ≤ 0.01. Data are presented as mean ± SEM.

We next investigated whether cholesterol depletion altered the lipid raft compartmentalization of proteins involved in DA release in striatal synaptosomes. Cholesterol content was measured using an Amplex Red Cholesterol assay following treatment with 0.3, 3, or 10 mM MβCD (Fig. 2A). A one-way ANOVA indicated a significant main effect of MβCD treatment, F_(3,16)_ = 5.80, p = 0.0070. Post hoc Tukey’s tests revealed reduced cholesterol at 3 mM (p = 0.039) and 10 mM (p = 0.040), but not 0.3 mM (p > 0.99). Notably, cholesterol decreased by ~40% at both 3 and 10 mM, suggesting that cholesterol depletion plateaued within this concentration range.

To determine whether MβCD disrupted membrane order, we measured Laurdan generalized polarization (GP) across different concentrations of MβCD: 0.3, 3, 10, and 50 mM (Fig. 2B). A one-way ANOVA revealed a significant effect of treatment on GP, F_(4,10)_ = 4.999, p = 0.0178. Tukey’s multiple-comparisons test showed that GP was significantly reduced only at 50 mM MβCD compared to control (p = 0.0098), whereas no significant differences were observed at 0.3, 3, or 10 mM (p ≥ 0.2211). These results indicate that MβCD reduced synaptosomal cholesterol at 3 and 10 mM without measurably disrupting overall membrane packing, whereas 50 mM MβCD produced a significant reduction in GP values, suggesting that the membrane is more fluid under this condition.

Lipid rafts are cholesterol-enriched membrane microdomains that assemble proteins for efficient signaling, and MβCD is widely used to perturb raft organization by extracting membrane cholesterol (Meza et al., 2023). We therefore tested whether 3 mM MβCD altered the association of proteins involved in exocytosis with raft and non-raft microdomains using sucrose density gradient ultracentrifugation of striatal synaptosomes. To define lipid raft and non-raft regions, we used flotillin, a well-established raft marker, and the transferrin receptor, a non-raft marker (Brown, 2006; Nothdurfter et al., 2007). Flotillin was present solely in fractions 4–6 and the transferrin receptor was present in fractions 13–15 (Fig. 2C). Therefore, fractions 4–6 were designated as raft fractions and fractions 13–15 as non-raft fractions, and these regions were used to determine the relative raft distribution of the proteins probed here.

Because MβCD reduced DA release, we asked whether this effect might be associated with altered raft localization of proteins involved in exocytosis. Vesicular neurotransmitter release, including DA release, is mediated by the assembly of the core SNARE complex consisting of syntaxin-1A, SNAP-25, and VAMP2 (Alexander et al., 2018; Chamberlain and Gould, 2002). Previous studies have shown that lipid rafts regulate SNARE-mediated exocytosis by coordinating the spatial organization of SNARE proteins (Chamberlain et al., 2001; Salaün et al., 2005a). Treatment with MβCD decreased the relative raft association of VAMP2 compared to vehicle-treated controls (Fig. 2F), t(6) = 2.983, p = 0.0245, with a large observed effect size, Cohen’s d = 2.11. Post hoc sensitivity analysis indicated that n = 4/group provides 80% power to detect only very large effects of Cohen’s d ≥ 2.38. MβCD did not significantly alter the relative raft association of syntaxin-1A (Fig. 2D), t(6) = 0.9173, p = 0.3944, or SNAP-25 (Fig. 2E), t(6) = 0.2265, p = 0.8284. We next examined synaptotagmin-1 and CaV2.2, key components of the calcium-dependent machinery that trigger synaptic vesicle fusion (Banerjee et al., 2020; Xia et al., 2007). Treatment with MβCD did not alter the relative raft association of synaptotagmin-1 (Fig. 2H), t(6) = 1.378, p = 0.2174, or CaV2.2 (Fig. 2G), t(6) = 0.9820, p = 0.3640. Cholesterol depletion could also have reduced DA reuptake by altering the raft compartmentalization of DAT. However, relative DAT raft association did not change following MβCD treatment (Fig. 2J), t(6) = 0.5470, p = 0.6041. Total expression of all measured proteins remained unchanged (Supplemental Fig. 3). Together, these data suggest that VAMP2 raft association is particularly sensitive to cholesterol depletion compared with the other proteins examined in this study.

### 3.3. MβCD decreased actin polymerization

Because MβCD decreased the raft association of VAMP2, a v-SNARE protein involved in vesicle docking and fusion, we next asked whether cholesterol depletion also disrupted the actin cytoskeleton, which coordinates synaptic vesicle trafficking and availability for exocytosis. Vesicle release depends on the dynamic balance between F-actin polymers and G-actin monomers. Polymerized F-actin helps organize vesicle pools and position vesicles near release sites, whereas the continuous turnover between F- and G-actin is required for vesicle mobilization, recruitment, and recycling during neurotransmitter release (Boiero Sanders et al., 2024). To test whether cholesterol depletion altered actin polymerization, we treated striatal synaptosomes and slices containing the NAc with 3 mM MβCD and quantified F-actin content.

**Fig. 3.**
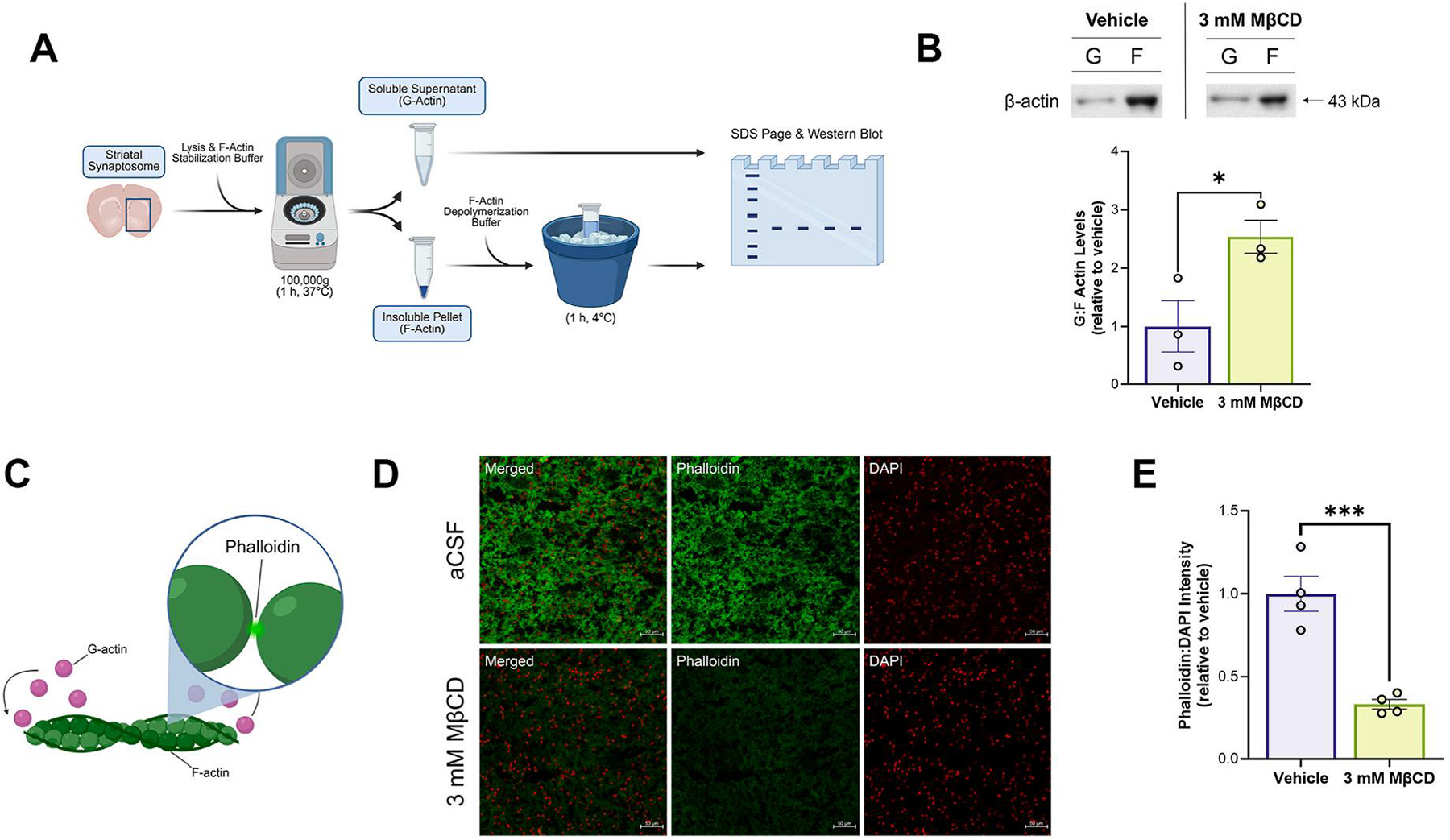
Effects of MβCD on actin polymerization. (A) Workflow for G-actin/F-actin fractionation in striatal synaptosomes. (B) Representative β-actin blots and quantification showing an increased G:F-actin ratio after 3 mM MβCD treatment (n = 3 animals/group). (C) Illustration of phalloidin binding to F-actin. (D) Confocal images of phalloidin (F-actin; green) and DAPI (nuclei; red) in NAc-containing slices treated with 3 mM MβCD compared to control. Scale bar: 50 μm. (E) Quantification of phalloidin intensity normalized to DAPI showing reduced F-actin levels following MβCD treatment. Points indicate slice means (n = 4 slices/group). Bars show mean ± SEM. *p ≤ 0.05, **p ≤ 0.01, ***p ≤ 0.001.

We first performed an actin fractionation assay in synaptosomes to separate G-actin from F-actin. Immunoblotting of β-actin, one of the major actin isoforms in the brain, revealed that MβCD increased the G:F-actin ratio, t(4) = 2.924, p = 0.0431, Cohen’s d = 2.39, corresponding to a ~2.54-fold shift toward monomeric G-actin (Fig. 3A–B). Post hoc sensitivity analysis indicated that n = 3/group provides 80% power to detect only very large effects of approximately Cohen’s d ≥ 3.07. As a complementary approach, we used phalloidin to visualize F-actin in NAc-containing slices treated with MβCD. Phalloidin is a fluorescent probe that binds a hydrophobic cleft formed between adjacent actin subunits along F-actin filaments (Fig. 3C). Phalloidin staining was reduced in MβCD-treated slices compared with control (Fig. 3D). Quantification of phalloidin intensity normalized to DAPI confirmed a marked reduction, t(6) = 6.086, p = 0.0009, Cohen’s d = 4.30, reflecting a ~3-fold lower phalloidin signal in the MβCD group (Fig. 3E). For the phalloidin assay, sensitivity analysis indicated that n = 4/group provides 80% power to detect effects of approximately Cohen’s d ≥ 2.38. These findings show that acute membrane cholesterol depletion decreased F-actin content and shifted the actin pool toward the monomeric (G-actin) state, which is consistent with destabilization of the actin cytoskeleton.

## 4. Discussion

The present study revealed four major findings. First, membrane cholesterol depletion by MβCD impaired electrically evoked DA release and reuptake, providing the first direct evidence in native striatal tissue that cholesterol simultaneously regulates both processes that govern striatal DA homeostasis. Second, cholesterol depletion reduced the ability of cocaine to inhibit DAT-mediated DA reuptake, consistent with the notion that cholesterol promotes the outward-facing conformation of DAT required for high-affinity ligand binding. Third, the reduction in DAT function did not appear to result from altered membrane compartmentalization of DAT or its known interacting partner syntaxin-1A, as neither protein showed a change in raft partitioning following cholesterol depletion. Finally, MβCD disrupted the lipid raft association of the vesicle-associated SNARE protein VAMP2 and reduced actin polymerization, suggesting that cholesterol depletion may dysregulate aspects of neurotransmitter vesicle trafficking and fusion that underlie DA release.

### 4.1. Cholesterol depletion disrupts DAT-mediated DA reuptake

We found that MβCD significantly reduced electrically evoked DA reuptake in striatal slices. This finding aligns with prior reports showing that MβCD (2.5–10 mM) impairs DAT function in HEK293 cells, N2A cells, EM4 cells, and striatal synaptosomes (Cremona et al., 2011; Hong and Amara, 2010; Jones et al., 2012; Rudin et al., 2026; Wang et al., 2023). Importantly, because Laurdan GP was unchanged at MβCD concentrations that impaired DA reuptake, these effects are likely not explained by nonspecific disruption of bulk membrane order. Structural, computational, and biochemical studies show that membrane cholesterol stabilizes the outward-facing DAT conformation, which is required for initial DA binding during transport (Hong and Amara, 2010; Penmatsa et al., 2013; Wang et al., 2023; Zeppelin et al., 2018). Cocaine preferentially binds to and stabilizes this outward-facing state and thus serves as a functional probe of the availability of DAT in this conformation (Beuming et al., 2008). Our finding that MβCD reduced the ability of cocaine to inhibit DA reuptake in striatal slices further supports the notion that cholesterol depletion reduces DAT function by destabilizing the outward-facing DAT conformation required for high-affinity DA reuptake. Alternatively, decreased DA reuptake could be due to reduced DAT surface availability. However, prior surface biotinylation experiments in HEK293 cells, LLC-PK_1_ cells, and rat striatal preparations have generally reported little to no reduction in basal surface DAT levels following MβCD treatment (Foster et al., 2008; Hong and Amara, 2010; Jones et al., 2012). While one study observed a modest (~16%) decrease in surface DAT in LLC-PK_1_ cells (Foster et al., 2008), the same treatment reduced DA uptake by 32%, suggesting that reduced surface DAT expression may not solely explain reduced DAT function.

We next determined whether DAT lipid raft association in striatal synaptosomes was altered. DAT was detected in both lipid raft and non-raft fractions, with the majority of DAT localized to non-raft regions. However, cholesterol depletion with MβCD did not alter DAT raft partitioning. To our knowledge, this is the first study in native striatal tissue to examine DAT raft localization following cholesterol depletion. Our findings are consistent with a previous study in LLC-PK_1_ cells showing no change in DAT raft association after 5 mM MβCD treatment using a Triton X-100-based fractionation (Foster et al., 2008). In contrast, other studies reported that 5 mM MβCD displaced DAT from raft fractions in HEK293 cells using a Brij-58-based fractionation (Hong and Amara, 2010; Jones et al., 2012). These inconsistencies likely reflect differences in model system, detergent extraction conditions, and baseline membrane cholesterol content (Hovde et al., 2019). More importantly, it has been demonstrated that disruption of DAT raft organization alone is insufficient to account for reduced DAT uptake, as treatment with nystatin, which disrupts lipid rafts but maintains cholesterol in the membrane, does not alter DAT-mediated DA uptake in HEK293 or EM4 cells (Cremona et al., 2011; Jones et al., 2012).

We also assessed whether MβCD altered the raft association of syntaxin-1A, which physically couples to DAT and modulates DA reuptake (Binda et al., 2008; Carvelli et al., 2008; Cervinski et al., 2010; Lee et al., 2004). However, syntaxin-1A was almost exclusively present in non-raft fractions in the present study, which is largely in agreement with prior literature in rat brain tissue and PC12 cells (Bacia et al., 2004; Hering et al., 2003; Lang, 2007; Lang et al., 2001). Importantly, syntaxin-1A raft localization was unchanged following cholesterol depletion, suggesting that syntaxin-1A did not play a role in MβCD-induced deficits in DA reuptake. Overall, these findings suggest that MβCD-induced reductions in DA reuptake are primarily attributed to alterations in DAT’s conformational state and are not explained by the redistribution of DAT or the DAT-interacting protein syntaxin-1A between lipid raft and non-raft microdomains. Future studies should determine whether cholesterol depletion alters the raft localization of other DAT interacting partners that modulate DAT-mediated uptake, such as protein kinase C, synaptogyrin-3, the DA D2 receptor, and σ-1 receptors (Hadlock et al., 2011; Sambo et al., 2018). Prior work has shown that σ1 receptors can interact with DAT and promote an outward-facing DAT conformation that facilitates cocaine binding (Hong et al., 2017). In addition, σ1 receptors can bind cholesterol and associate with lipid rafts (Palmer et al., 2007); therefore, cholesterol depletion may disrupt σ1 receptor-dependent regulation of DAT. Cholesterol depletion could also alter DAT phosphorylation at sites such as the N-terminal Thr53 residue, which regulates DAT-mediated uptake and cocaine binding (Challasivakanaka et al., 2017; Foster et al., 2012). Thr53 is a substrate for phosphorylation by MAPK family kinases, which can be activated by receptor tyrosine kinases to modulate DAT-mediated uptake (Gorentla et al., 2009; Hoover et al., 2007). Because receptor tyrosine kinases and downstream signaling complexes can be organized within lipid rafts at the plasma membrane (Domenichini et al., 2025), cholesterol depletion could alter this kinase membrane localization and disrupt MAPK signaling, leading to changes in DAT phosphorylation and reuptake function.

### 4.2. Cholesterol depletion reduces evoked DA release

Cholesterol depletion with MβCD reduced electrically evoked DA release in NAc slices, showing for the first time that membrane cholesterol depletion impairs DA release in native striatal tissue. To our knowledge, only two prior studies in PC12 cells have directly examined the effects of cholesterol depletion on DA release. One study found that MβCD (15 Mm, 30 min) reduced K^+^-evoked DA release (Lang et al., 2001), and another similarly found that treatment with lovastatin, a cholesterol synthesis inhibitor, combined with 5 mM MβCD decreased ATP-stimulated [^3^H]DA release (Chamberlain et al., 2001). Similar effects have been reported for glutamate release in brain tissue (Choi et al., 2015; Gil et al., 2005; Teixeira et al., 2012). For example, MβCD (1–10 mM, 10 min) reduced KCl-evoked glutamate release in rat cortical synaptosomes (Teixeira et al., 2012). Moreover, evoked glutamate release was reduced by ~70% in cultured hippocampal neurons from Niemann-Pick type C1-deficient mice, in which improper cholesterol trafficking reduces cholesterol content at synaptic terminals (Wasser et al., 2007). Crucially, this deficit was rescued by exogenous cholesterol addition, demonstrating a direct causal role for cholesterol in glutamate release. Collectively, prior work demonstrates that membrane cholesterol is an essential regulator of evoked neurotransmitter release, and our study extends these findings to striatal DA exocytosis in intact tissue. It is also important to note that membrane cholesterol depletion may influence postsynaptic mechanisms that regulate extracellular DA tone. For example, postsynaptic D2 receptor signaling can feed back onto DA terminals to modulate release (Anzalone et al., 2012). Thus, cholesterol depletion could potentially alter postsynaptic D2 receptor signaling and thereby indirectly affect feedback mechanisms that regulate presynaptic DA release, which warrants further investigation.

To identify the mechanisms underlying MβCD-induced reductions in DA release, we first examined whether cholesterol depletion altered the lipid raft localization of proteins involved in Ca^2+^-triggered exocytosis. At baseline, CaV2.2 channels primarily localized to raft fractions, whereas synaptotagmin-1 only partially associated with rafts, consistent with prior reports in synaptosomes and heterologous expression systems (Gil et al., 2005; Jia et al., 2006; Robinson et al., 2010; Ronzitti et al., 2014). Neither protein showed redistribution following MβCD treatment, suggesting that altered raft association of exocytosis steps beyond Ca^2+^ entry may better explain the effects of cholesterol depletion on DA release.

Because assembly of the core SNARE complex is required for synaptic vesicle fusion and DA release (Fortin et al., 2006), we next assessed whether MβCD altered the raft association of SNARE proteins. SNAP-25 showed strong raft partitioning at baseline, whereas syntaxin-1A localized predominantly to non-raft fractions, and VAMP2 exhibited modest raft association. This heterogeneous distribution is consistent with previous studies in synaptosomes and neuronal cell lines (Bacia et al., 2004; Gil et al., 2006; Greaves et al., 2010; Jia et al., 2006; Lang, 2007; Salaün et al., 2005b). Following MβCD treatment, the raft association of SNAP-25 and syntaxin-1A was unchanged; however, VAMP2 partially translocated out of raft fractions. This observation suggests that the membrane organization of the vesicle-associated v-SNARE VAMP2 is more vulnerable to cholesterol depletion than that of the plasma membrane-associated t-SNAREs syntaxin-1A and SNAP-25. Given that synaptic vesicles are highly enriched in cholesterol, disrupted organization of vesicle-associated VAMP2 could impair SNARE complex assembly and thereby contribute to MβCD-induced reductions in release. The sensitivity of VAMP2 to cholesterol depletion is supported by prior studies. For example, MβCD reduced evoked glutamate release in mouse hippocampal neurons of wild-type mice, but not of VAMP2 knockout mice, suggesting that cholesterol depletion impairs a VAMP2-dependent step of exocytosis (Wasser et al., 2007). Further, in reconstituted vesicles, VAMP2 has been demonstrated to adopt conformations that favor SNARE complex assembly in raft-like environments, whereas non-raft environments reduce VAMP2’s availability for assembly (Wang et al., 2020). Taken together, even a partial loss of raft-associated VAMP2 could impair the formation of SNARE complexes and reduce the efficiency of evoked vesicular exocytosis.

Reduced DA release by MβCD could also result from disruption of the actin cytoskeleton. The dynamic cycling between F- and G-actin organizes vesicle pools and provides structural tracks that mobilize vesicles toward release sites, and F-actin further mediates synaptic vesicle positioning and fusion (Morales et al., 2000; Nolan et al., 2020; Zhang and Augustine, 2021). Disruption of actin polymerization has been shown to reduce presynaptic glutamate release probability in rat hippocampal slices (Kim and Lisman, 1999). Actin filaments are also closely linked to membrane cholesterol, as cholesterol-rich lipid rafts are physically and functionally coupled to F-actin pools that help maintain membrane organization and compartmentalization (Biswas et al., 2019; Chichili and Rodgers, 2009; Cubí et al., 2013). Therefore, cholesterol depletion could disrupt actin dynamics by altering the association between the actin cytoskeleton and lipid rafts. In the current study, treatment of both synaptosomes and striatal slices with MβCD reduced actin polymerization, as indicated by an increased G:F-actin ratio and reduced phalloidin labeling, respectively. Previous reports have similarly shown that treatment with MβCD (5 mM, 20 min) reduced phalloidin labeling in hippocampal neurons, which was reversed by pharmacological stabilization of actin polymerization (Hering et al., 2003). To directly test whether actin depolymerization contributes to MβCD-induced DA release deficits, we attempted to stabilize actin with jasplakinolide following MβCD treatment in slices. However, jasplakinolide alone further reduced evoked DA release, making rescue experiments difficult to interpret (Supplemental Fig. 4). This suggests that disruption of actin polymerization in either direction can disrupt evoked DA release. Our finding that MβCD reduced the raft association of VAMP2 may also be relevant to the observed effects of cholesterol depletion on actin dynamics, as VAMP2 is located on synaptic vesicles, and F-actin mobilizes those vesicles to release sites at the plasma membrane. These two findings may therefore not be independent. Cholesterol depletion may reduce DA release, in part, by disrupting actin polymerization and thereby impairing vesicle trafficking and positioning at release sites. At the same time, the partial displacement of VAMP2 from lipid rafts suggests that vesicles that reach the membrane may also be less efficiently organized for SNARE complex assembly and fusion.

Importantly, MβCD altered VAMP2 raft association and actin polymerization, which are shared presynaptic mechanisms involved in vesicle trafficking and release across many neurotransmitter systems, suggesting that MβCD’s effects are unlikely to be specific to DA signaling. However, cholesterol depletion does not appear to affect all neurotransmitter systems equally. Consistent with this, 2 mM MβCD for 30 min reduced DAT-mediated uptake in HEK293 cells without significantly altering serotonin or norepinephrine transporter-mediated uptake, whereas higher concentrations impaired uptake across all three transporters (Rudin et al., 2026). In line with this, cholesterol depletion differentially alters the function and localization of some G protein-coupled receptors, as ~15 mM MβCD for 1 h selectively impaired μ-opioid, but not δ-opioid receptor function in HEK293 cells (Levitt et al., 2009). Therefore, it is of interest to determine whether cholesterol depletion in native tissue differentially alters neurotransmitter release across distinct neurotransmitter systems. Further, we selected 3 mM MβCD for downstream assays because it produced deficits in DA release and reuptake and reduced free cholesterol in synaptosomes by ~40%, without significantly altering membrane order, as measured by Laurdan GP. However, while Laurdan GP provides a useful measure of overall membrane packing, this assay may not detect subtle changes in membrane organization. Complementary approaches, such as DPH fluorescence anisotropy, which measures membrane fluidity from a deeper region of the lipid bilayer (Morissette et al., 2018), or di-4-ANEPPDHQ imaging, which distinguishes more ordered, raft-like membrane environments from less ordered, non-raft membrane environments (Jin et al., 2005), could provide a more comprehensive assessment of how MβCD alters membrane organization in synaptosomes. Importantly, changes in cholesterol of similar magnitudes have been previously reported in human neurological disease. For example, a 36% reduction in membrane cholesterol was observed in hippocampal samples from Alzheimer’s disease patients carrying the ApoE4 allele (Ledesma et al., 2003).

It is important to acknowledge that MβCD can also affect other membrane lipids, including phospholipids and sphingolipids. For example, in cultured rat cerebellar granule neurons, 5 mM MβCD for 30 min depleted 50.2% of cholesterol, while also decreasing sphingomyelin, glycosphingolipids, and glycerophospholipids by 2-17% (Ottico et al., 2003). In addition, although MβCD significantly reduced actin polymerization in the present study, other components of vesicle trafficking machinery, such as microtubules, motor proteins, and membrane-associated small GTPases could also be affected and merit further investigation. Moreover, because MβCD was not expected to be able to penetrate thick brain slices evenly, we did not directly validate cholesterol depletion in the slices used for FSCV. Instead, cholesterol content was measured in synaptosomes treated with the same concentrations of MβCD. Because FSCV primarily measures DA transmission on the surface of the slice surrounding the recording electrode, future studies could address this more directly by using thinner slices and measuring cholesterol on more superficial regions with filipin or genetically encoded D4H-based cholesterol sensors (Maekawa, 2017; Sun et al., 2014). Additionally, only male rats were used for all experiments. Although no studies, to our knowledge, have directly compared cholesterol homeostasis between sexes, some studies indirectly indicate that sex differences may exist. For example, PET imaging showed greater striatal binding of a radiolabeled tracer for CYP46A1, a primary brain cholesterol catabolism enzyme, in women compared with men (Haider et al., 2022). This finding suggests that brain cholesterol turnover may differ by sex and that acute cholesterol depletion could have sex-dependent effects on DA terminal function. Future studies should determine whether cholesterol depletion similarly alters DA transmission and neurotransmitter release machinery in females.

### 4.3. Conclusions

Cholesterol depletion impaired DA release, DAT-mediated DA reuptake, and cocaine inhibition of DA reuptake in native striatal tissue, establishing membrane cholesterol as a key regulator of both major processes that govern overall DA terminal function. The reduction in DAT-dependent DA reuptake is most consistent with destabilization of the outward-facing DAT conformation, whereas impaired evoked DA release may be explained by disrupted raft compartmentalization of the vesicle-associated protein VAMP2 and depolymerization of the actin cytoskeleton. Because dysregulated brain cholesterol metabolism is present in Huntington’s disease, Parkinson’s disease, schizophrenia, and substance use disorders, all of which involve aberrant DA signaling, the mechanisms identified in the present study may have broad relevance for understanding how membrane lipid disturbances contribute to DA dysfunction in these conditions.

## Supplementary Material

Supplementary Material 1

Supplementary Material 2

## Figures and Tables

**Table 1:** Antibodies used for western blotting.

Target (Source)	Molecular Weight (kDa)	Supplier	Identifier	Dilution
β-actin (mouse)	42	Santa Cruz	47778	1:1000
CaV2.2 (Rabbit)	190	Cell Signaling	35175	1:200
Dopamine Transporter (Rabbit)	78	Millipore	AB15191	1:1000
Flotillin (Mouse)	47	Santa Cruz	Sc-74566	1:250
GAPDH (Mouse)	37	Santa Cruz	365062	1:1000
Goat anti-mouse IgG-HRP	-	Cell Signaling	7076	1:4000
Goat anti-rabbit IgG-HRP	-	Cell Signaling	7074	1:4000
Syntaxin-1A (Rabbit)	33	Cell Signaling	18572	1:1000
Synaptotagmin-1 (Rabbit)	60	Cell Signaling	14558	1:1000
SNAP-25 (Rabbit)	25	Cell Signaling	5308	1:1000
Transferrin Receptor (Mouse)	80	Invitrogen	13–6800	1:1000
VAMP2 (Rabbit)	13	Cell Signaling	13508	1:1000

## Data Availability

The data supporting the findings of this study are available from the corresponding author upon reasonable request.

## Appendix A. Supplementary data

Supplementary data to this article can be found online at https://doi.org/10.1016/j.neuropharm.2026.111078.

## Abbreviations

Ca^2+^: Calcium
CaV2.2: N-type voltage-gated calcium channel
DA: Dopamine
DAT: Dopamine transporter
DMF: N,N-Dimethylformamide
FSCV: Fast-scan cyclic voltammetry
GP: Generalized polarization
MβCD: Methyl-β-cyclodextrin
NAc: Nucleus accumbens
SNAP-25: Synaptosomal-associated protein of 25 kDa
SNARE: Soluble N-ethylmaleimide-sensitive factor attachment protein receptor
TBST: Tris-buffered saline containing 1% Tween-20
VAMP2: Vesicle-associated membrane protein 2
