# Supplementary material for: Disruption of dopamine transmission by cholesterol depletion is associated with alterations in protein lipid raft partitioning and actin dynamics
Source: Neuropharmacology. Author manuscript; Available in PMC 2026 Jul 1. (PMC13316772; doi:10.1016/j.neuropharm.2026.111078)
Supplement: Supplementary Material 1 [file NIHMS2190795-supplement-Supplementary_Material_1.pptx]

## Slide 1
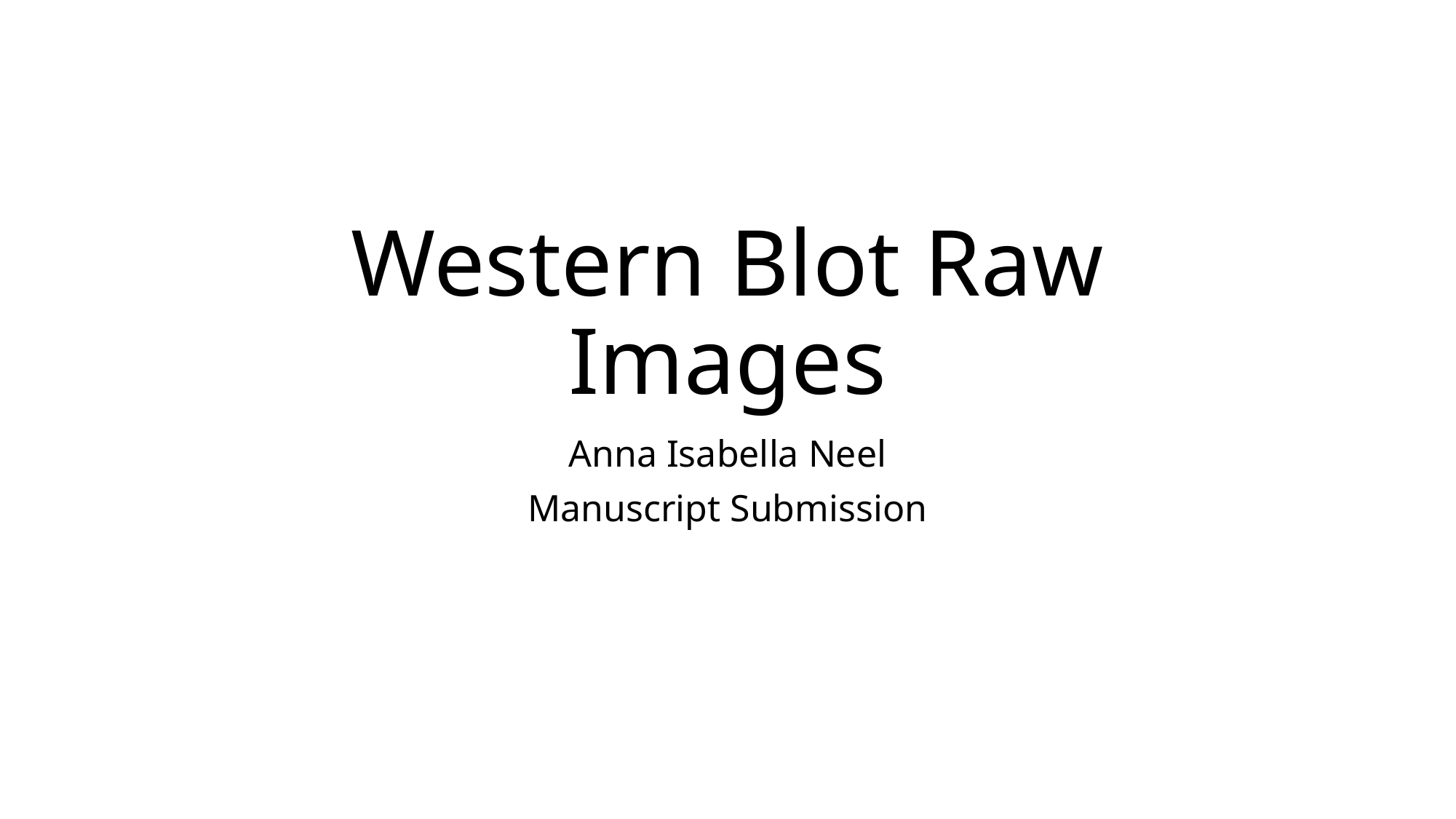

# Western Blot Raw Images
Anna Isabella Neel
Manuscript Submission

## Slide 2
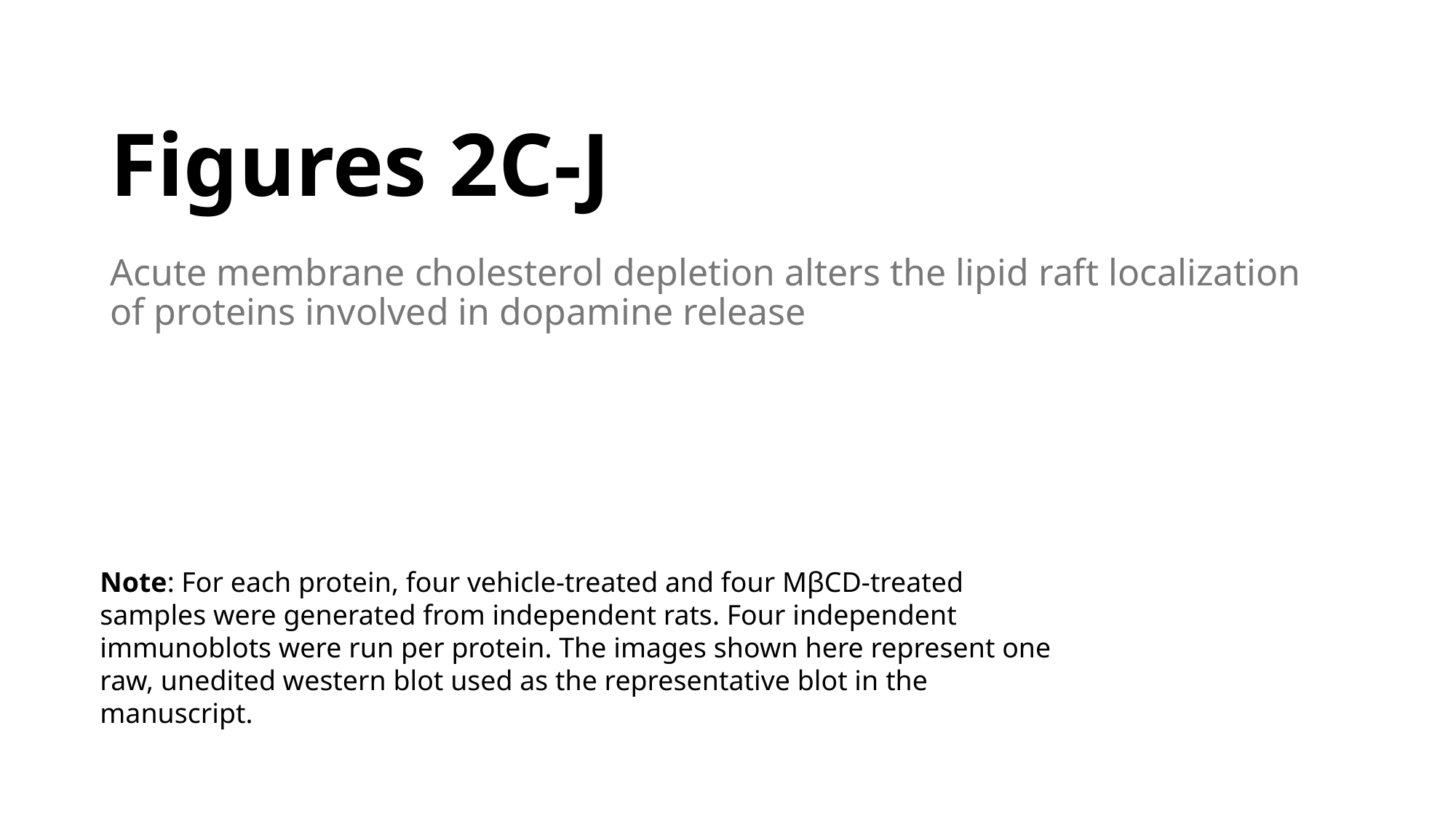

# Figures 2C-J
Acute membrane cholesterol depletion alters the lipid raft localization of proteins involved in dopamine release
Note: For each protein, four vehicle-treated and four MβCD-treated samples were generated from independent rats. Four independent immunoblots were run per protein. The images shown here represent one raw, unedited western blot used as the representative blot in the manuscript.

## Slide 3
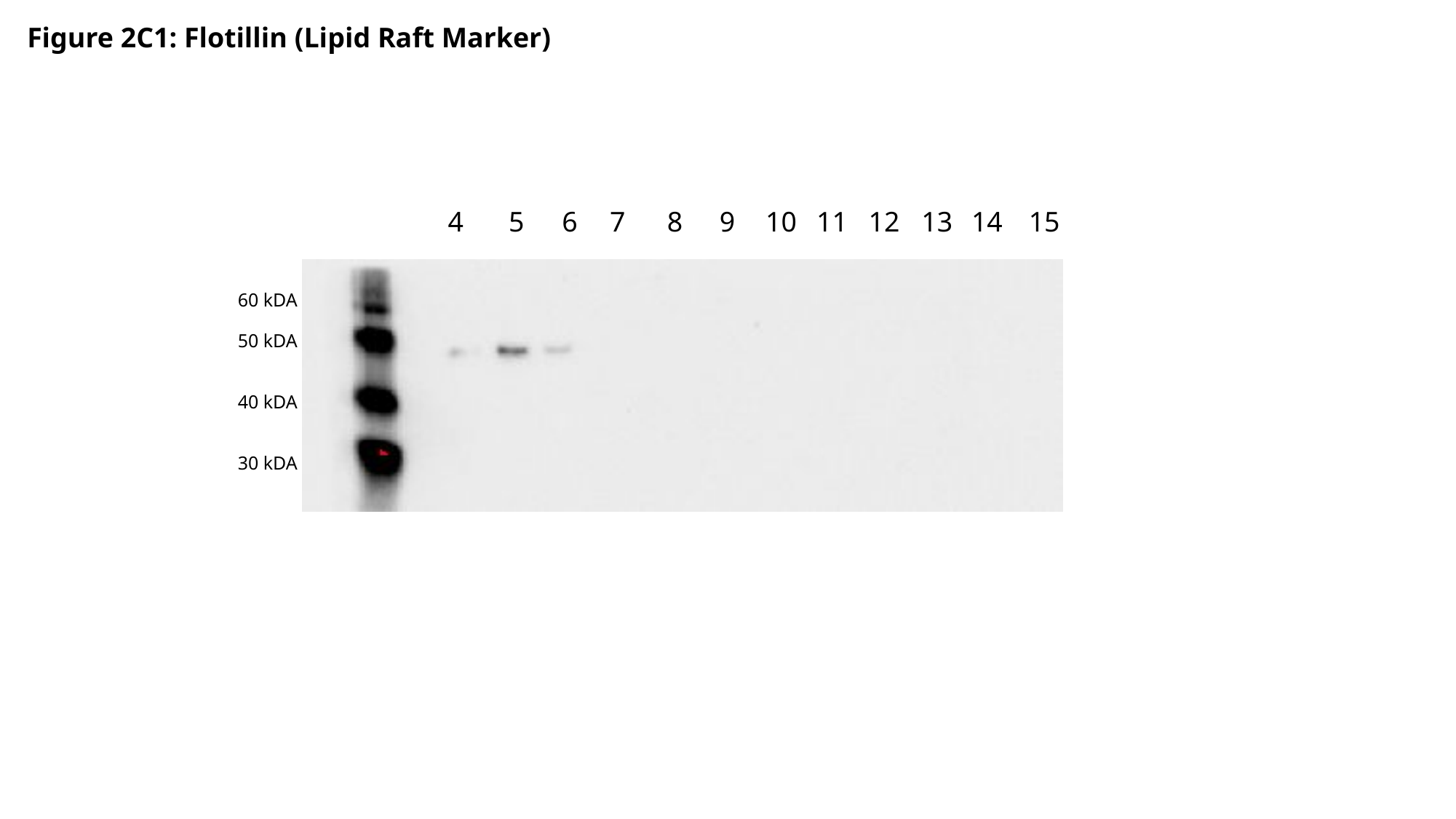

Figure 2C1: Flotillin (Lipid Raft Marker)
4
5
6
7
8
9
10
11
12
13
14
15
60 kDA
50 kDA
40 kDA
30 kDA

## Slide 4
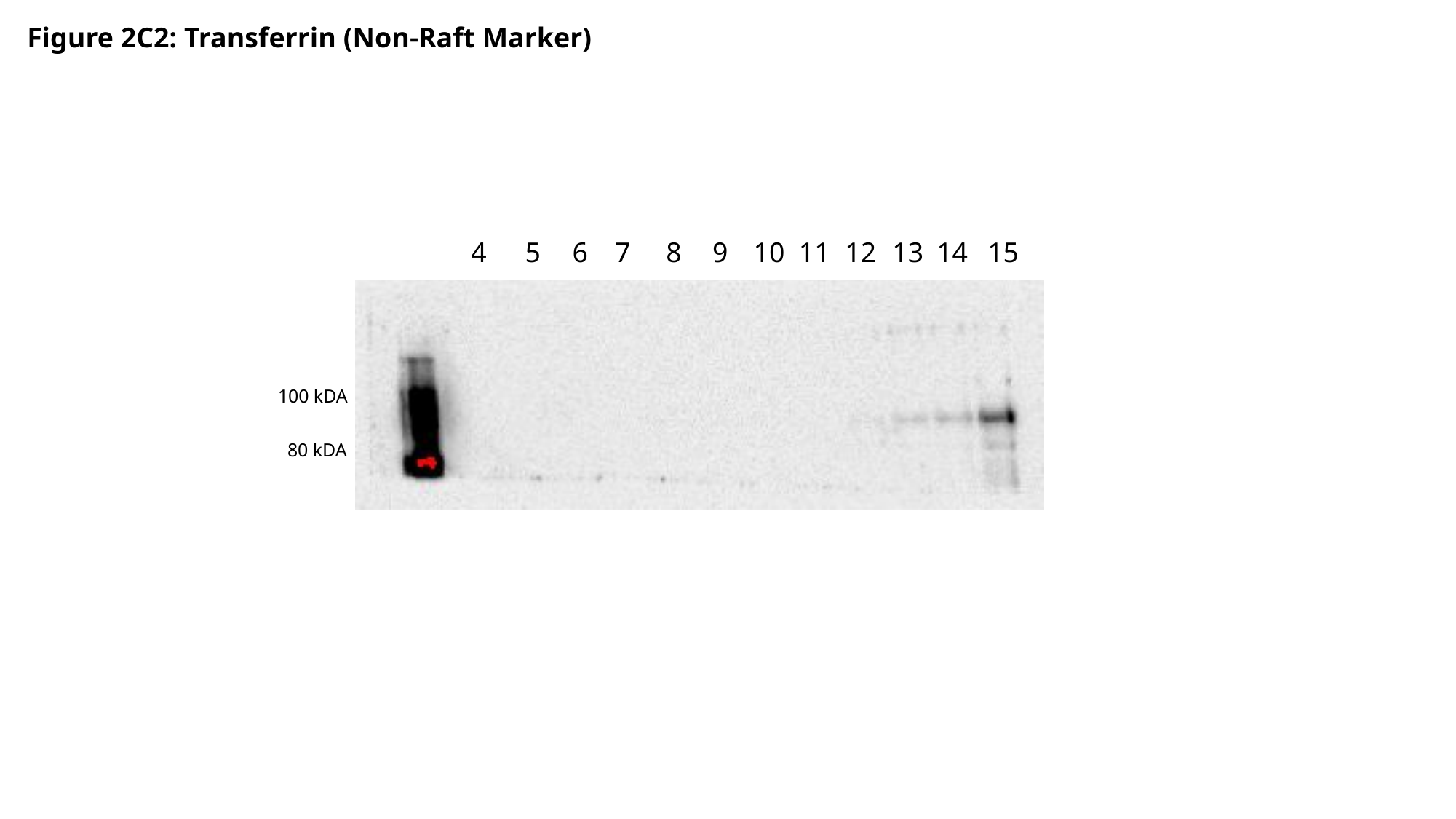

Figure 2C2: Transferrin (Non-Raft Marker)
4
5
6
7
8
9
10
11
12
13
14
15
100 kDA
80 kDA

## Slide 5
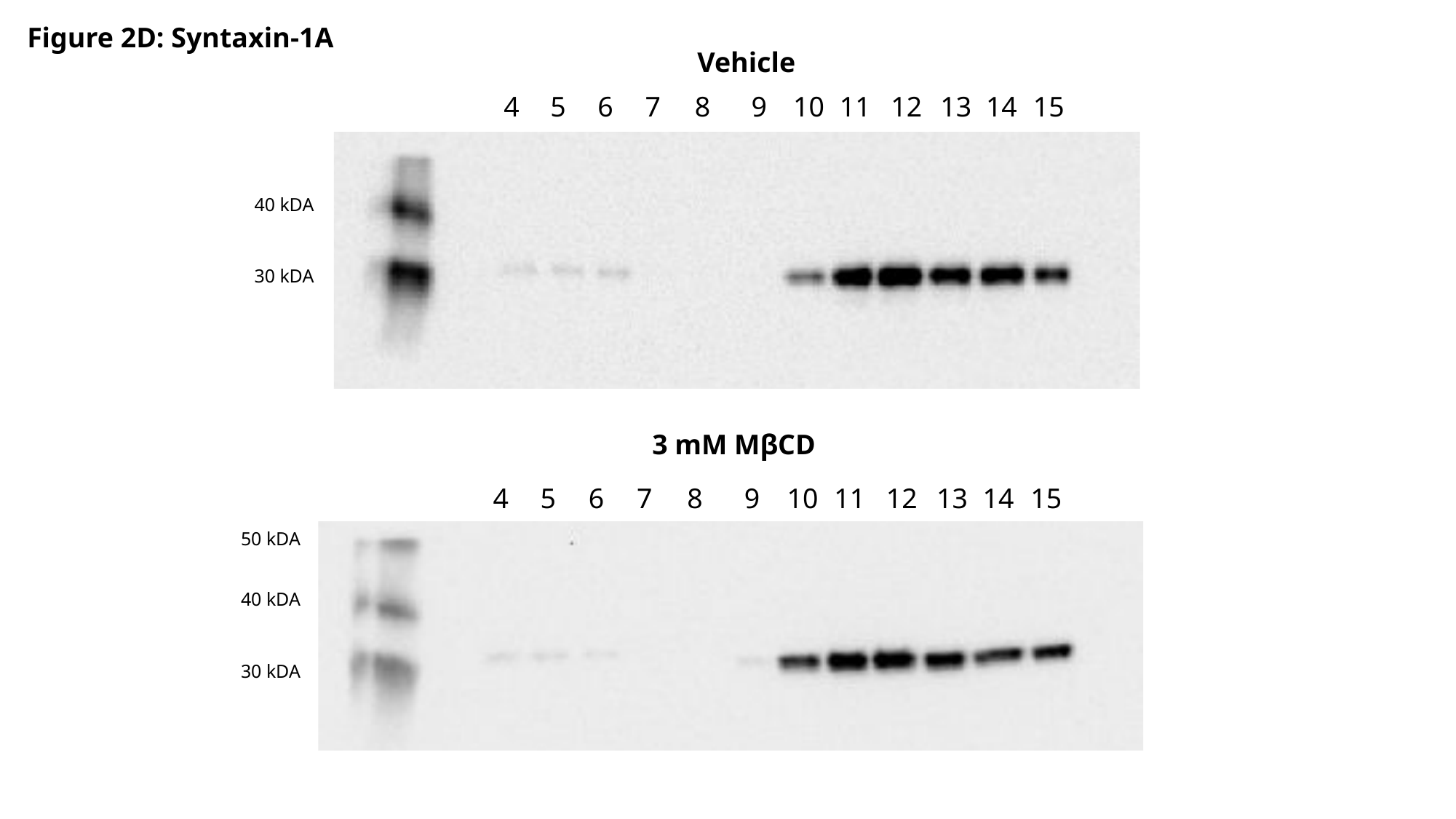

Figure 2D: Syntaxin-1A
Vehicle
4
5
6
7
8
9
10
11
12
13
14
15
40 kDA
30 kDA
3 mM MβCD
4
5
6
7
8
9
10
11
12
13
14
15
50 kDA
40 kDA
30 kDA

## Slide 6
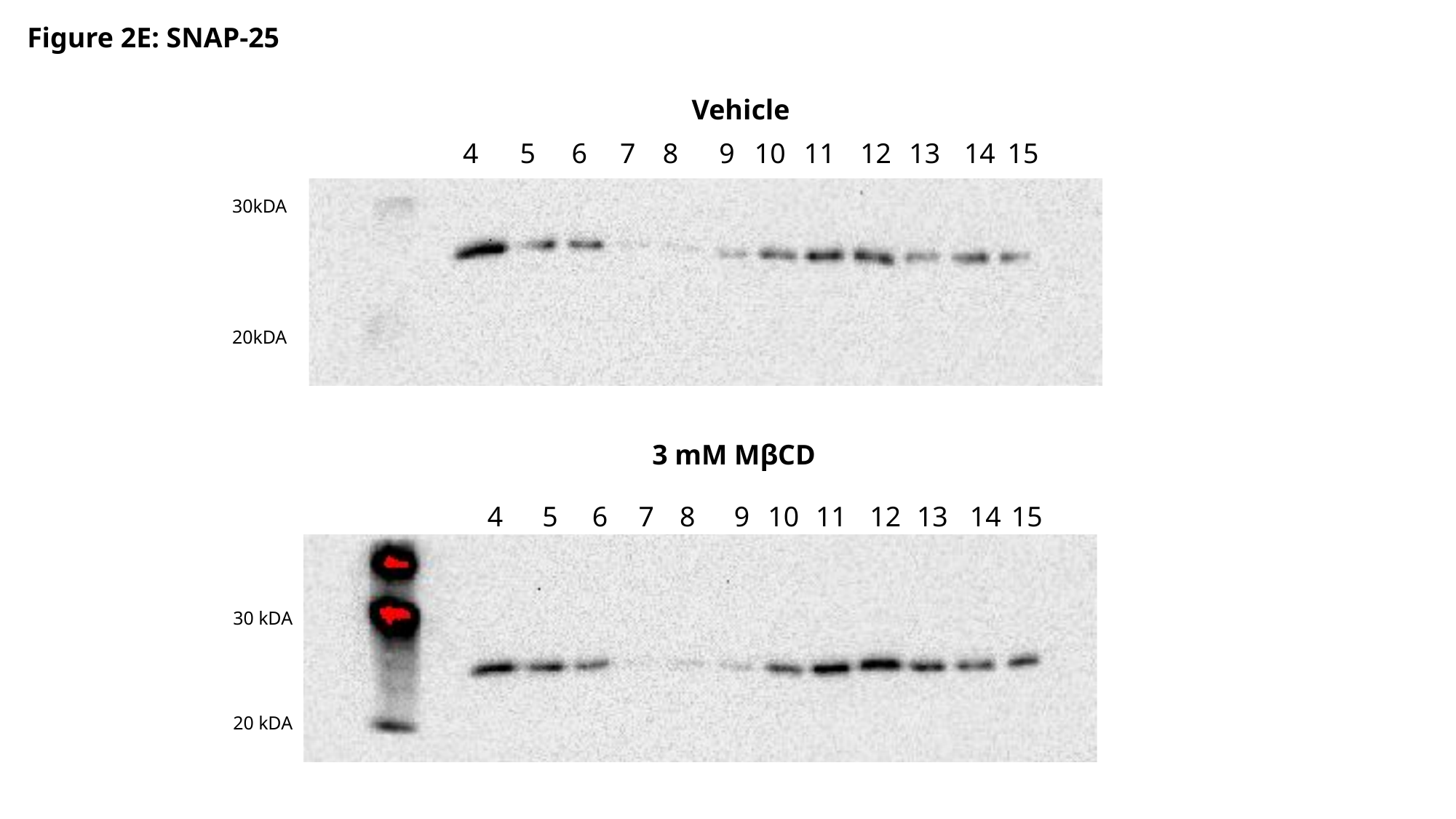

Figure 2E: SNAP-25
Vehicle
4
5
6
7
8
9
10
11
12
13
14
15
30kDA
20kDA
3 mM MβCD
4
5
6
7
8
9
10
11
12
13
14
15
30 kDA
20 kDA

## Slide 7
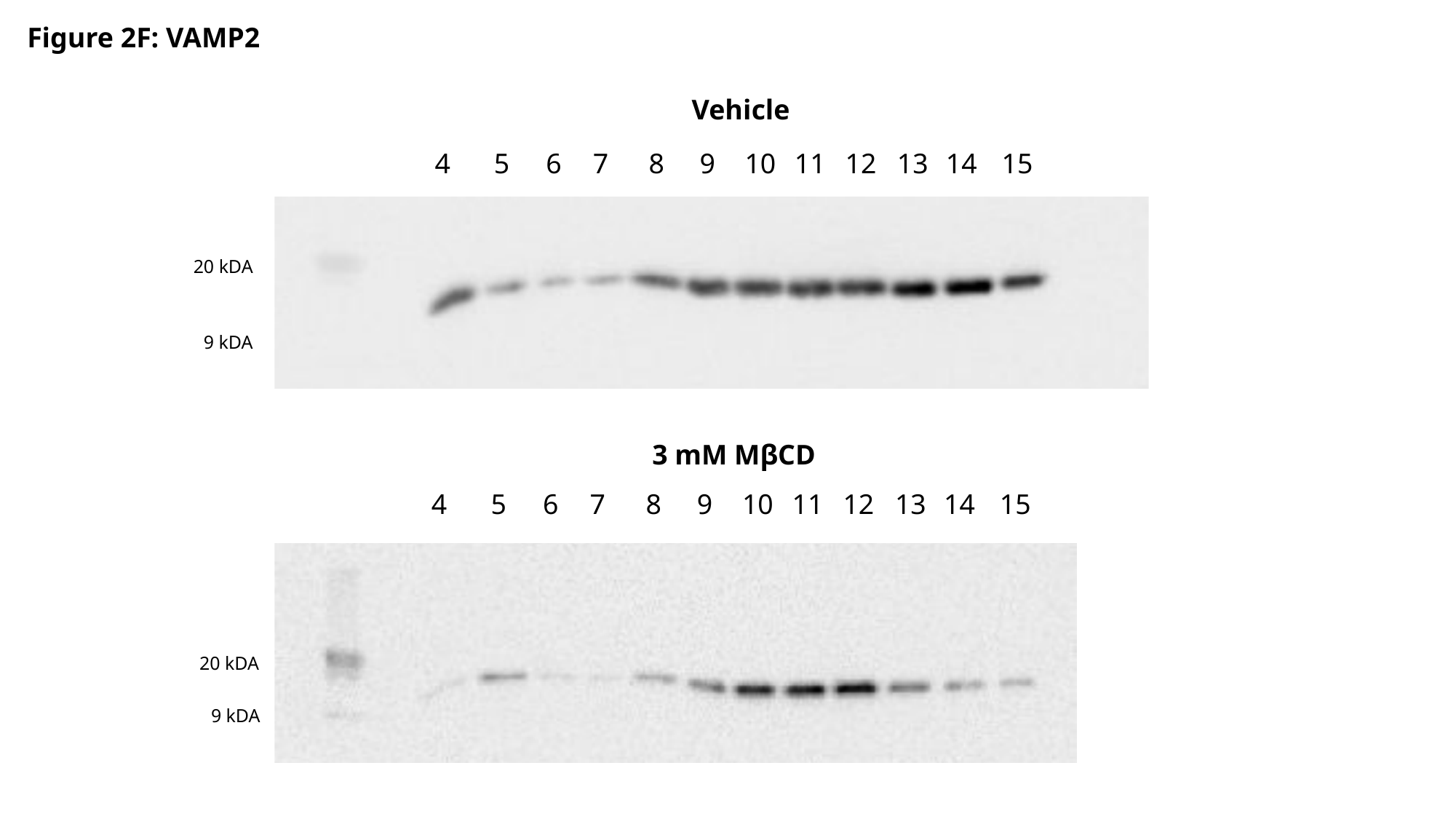

Figure 2F: VAMP2
Vehicle
4
5
6
7
8
9
10
11
12
13
14
15
20 kDA
9 kDA
3 mM MβCD
4
5
6
7
8
9
10
11
12
13
14
15
20 kDA
9 kDA

## Slide 8
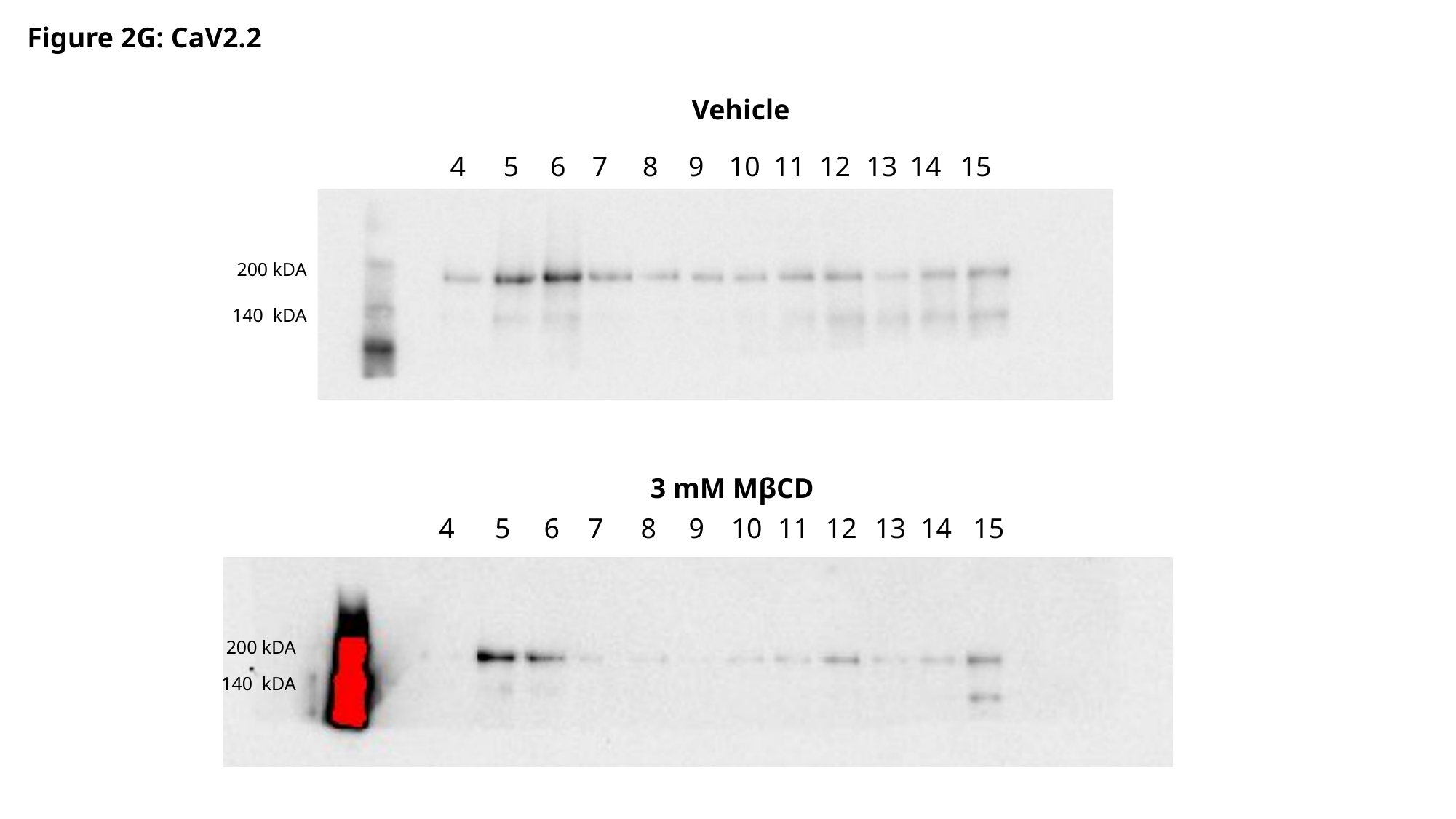

Figure 2G: CaV2.2
Vehicle
4
5
6
7
8
9
10
11
12
13
14
15
200 kDA
140 kDA
3 mM MβCD
4
5
6
7
8
9
10
11
12
13
14
15
200 kDA
140 kDA

## Slide 9
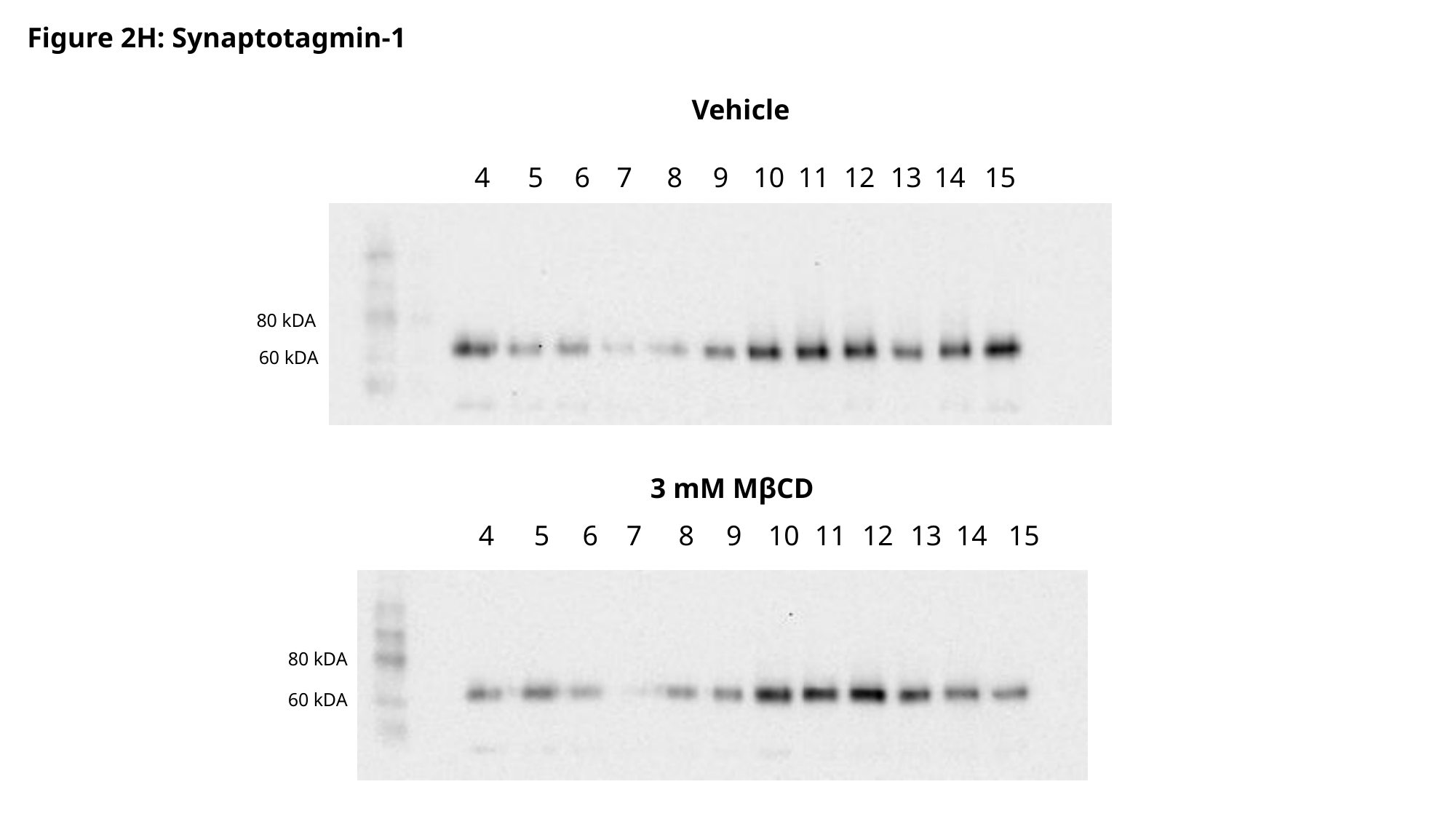

Figure 2H: Synaptotagmin-1
Vehicle
4
5
6
7
8
9
10
11
12
13
14
15
80 kDA
60 kDA
3 mM MβCD
4
5
6
7
8
9
10
11
12
13
14
15
80 kDA
60 kDA

## Slide 10
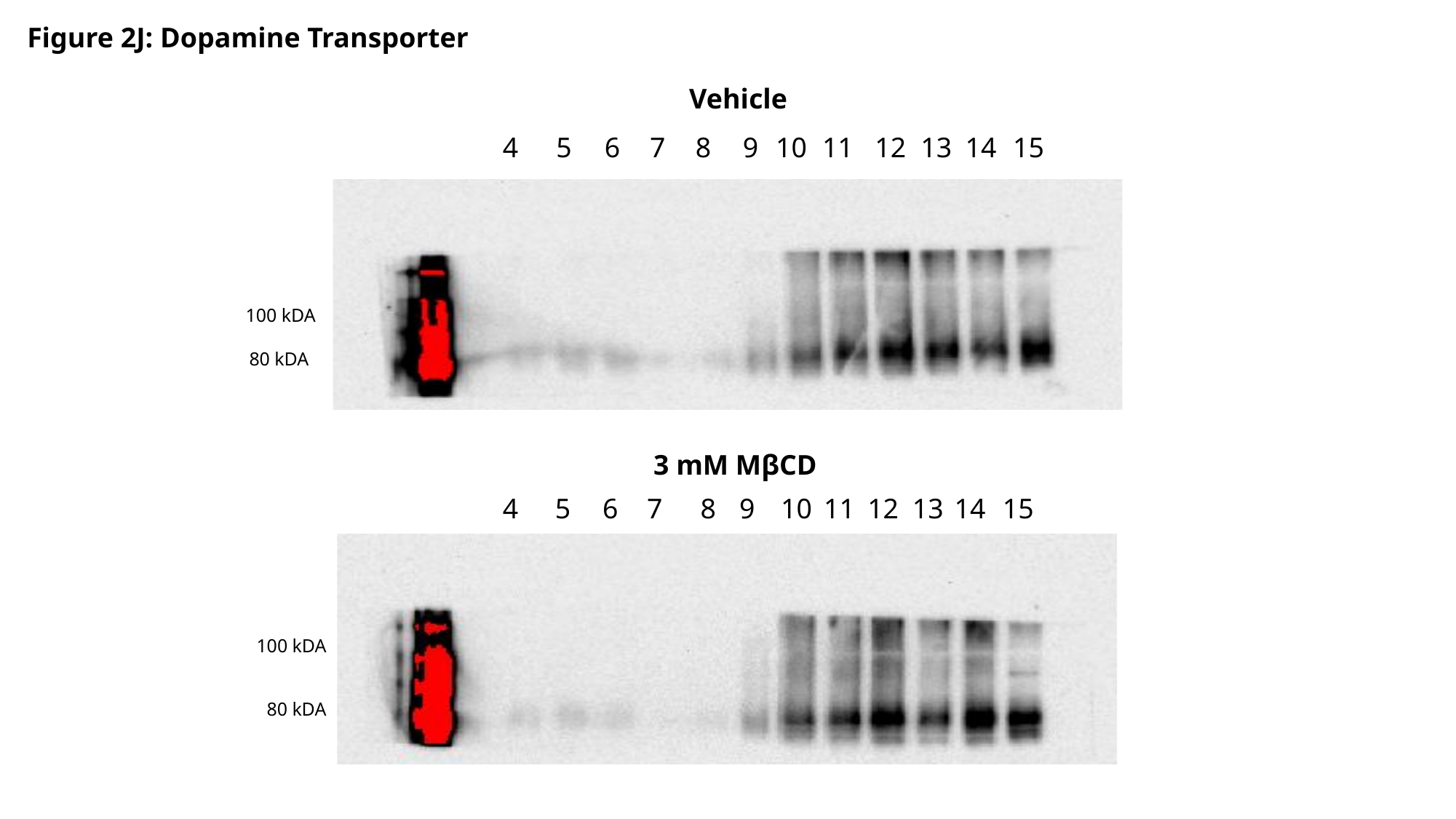

Figure 2J: Dopamine Transporter
Vehicle
4
5
6
7
8
9
10
11
12
13
14
15
100 kDA
80 kDA
3 mM MβCD
4
5
6
7
8
9
10
11
12
13
14
15
100 kDA
80 kDA

## Slide 11
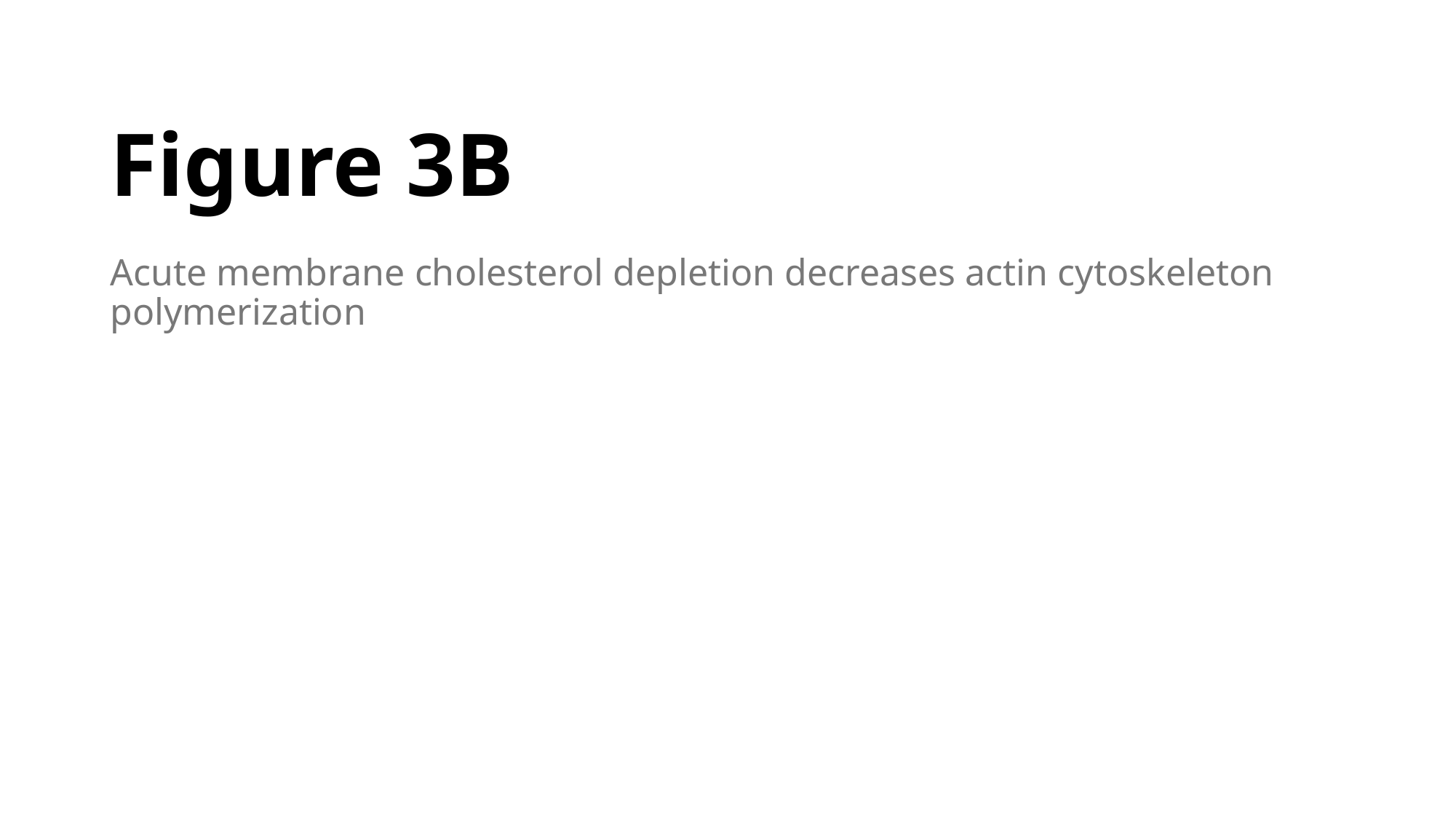

# Figure 3B
Acute membrane cholesterol depletion decreases actin cytoskeleton polymerization

## Slide 12
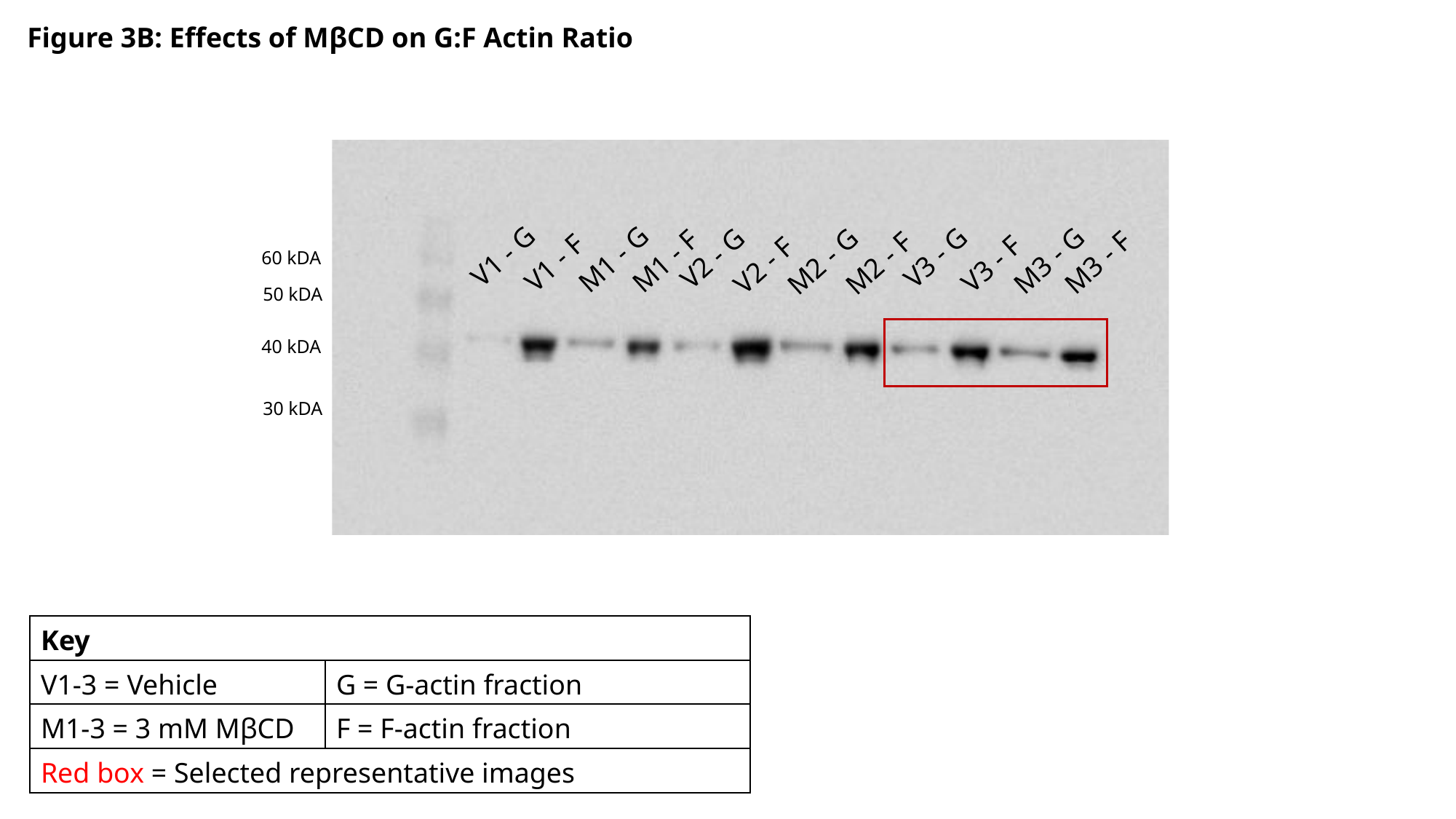

Figure 3B: Effects of MβCD on G:F Actin Ratio
M1 - F
V1 - F
M1 - G
V1 - G
M3 - F
V3 - F
M3 - G
V3 - G
M2 - F
V2 - F
M2 - G
V2 - G
60 kDA
50 kDA
40 kDA
30 kDA
| Key | |
| --- | --- |
| V1-3 = Vehicle | G = G-actin fraction |
| M1-3 = 3 mM MβCD | F = F-actin fraction |
| Red box = Selected representative images | |

## Slide 13
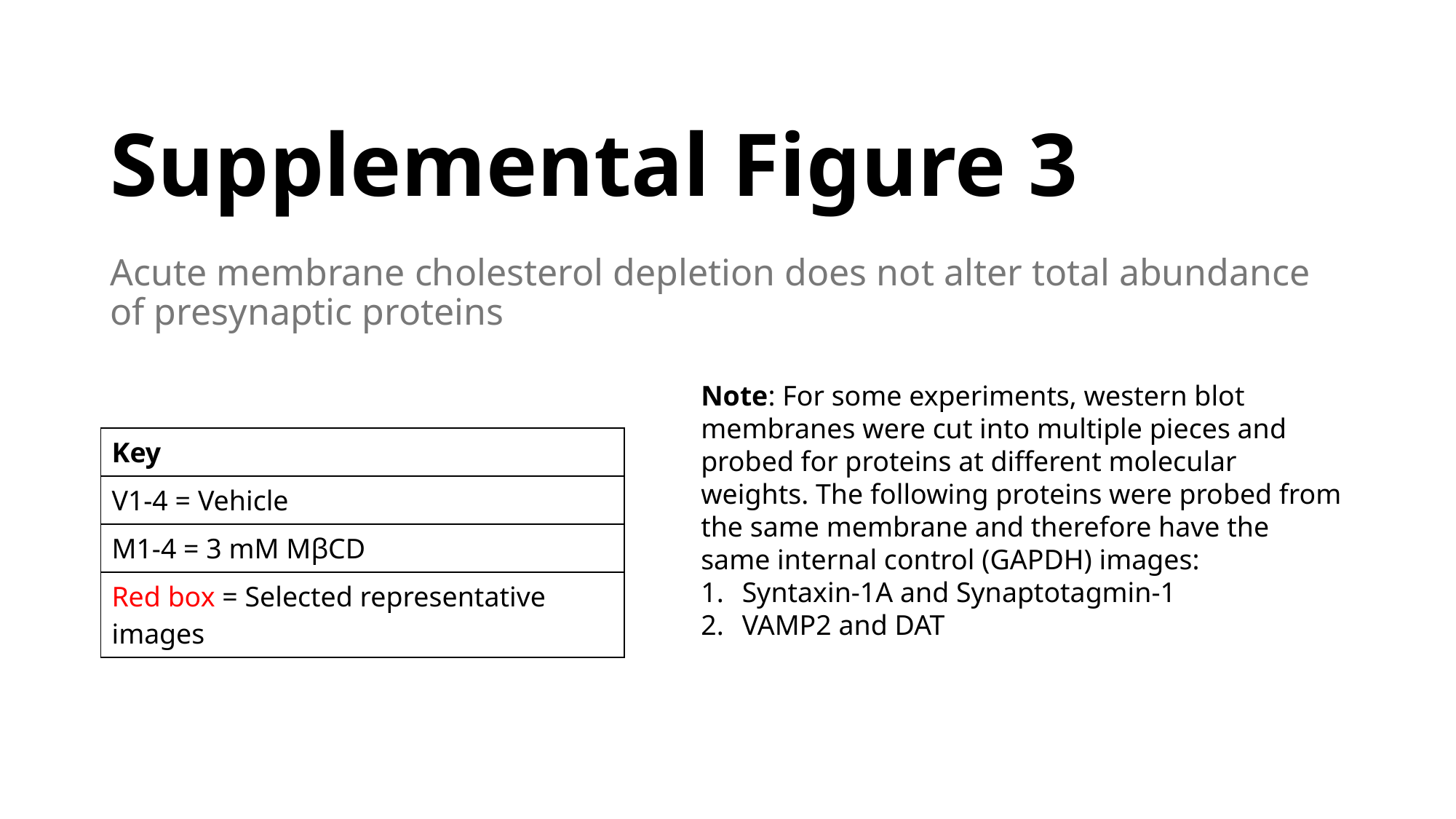

# Supplemental Figure 3
Acute membrane cholesterol depletion does not alter total abundance of presynaptic proteins
Note: For some experiments, western blot membranes were cut into multiple pieces and probed for proteins at different molecular weights. The following proteins were probed from the same membrane and therefore have the same internal control (GAPDH) images:
Syntaxin-1A and Synaptotagmin-1
VAMP2 and DAT
| Key |
| --- |
| V1-4 = Vehicle |
| M1-4 = 3 mM MβCD |
| Red box = Selected representative images |

## Slide 14
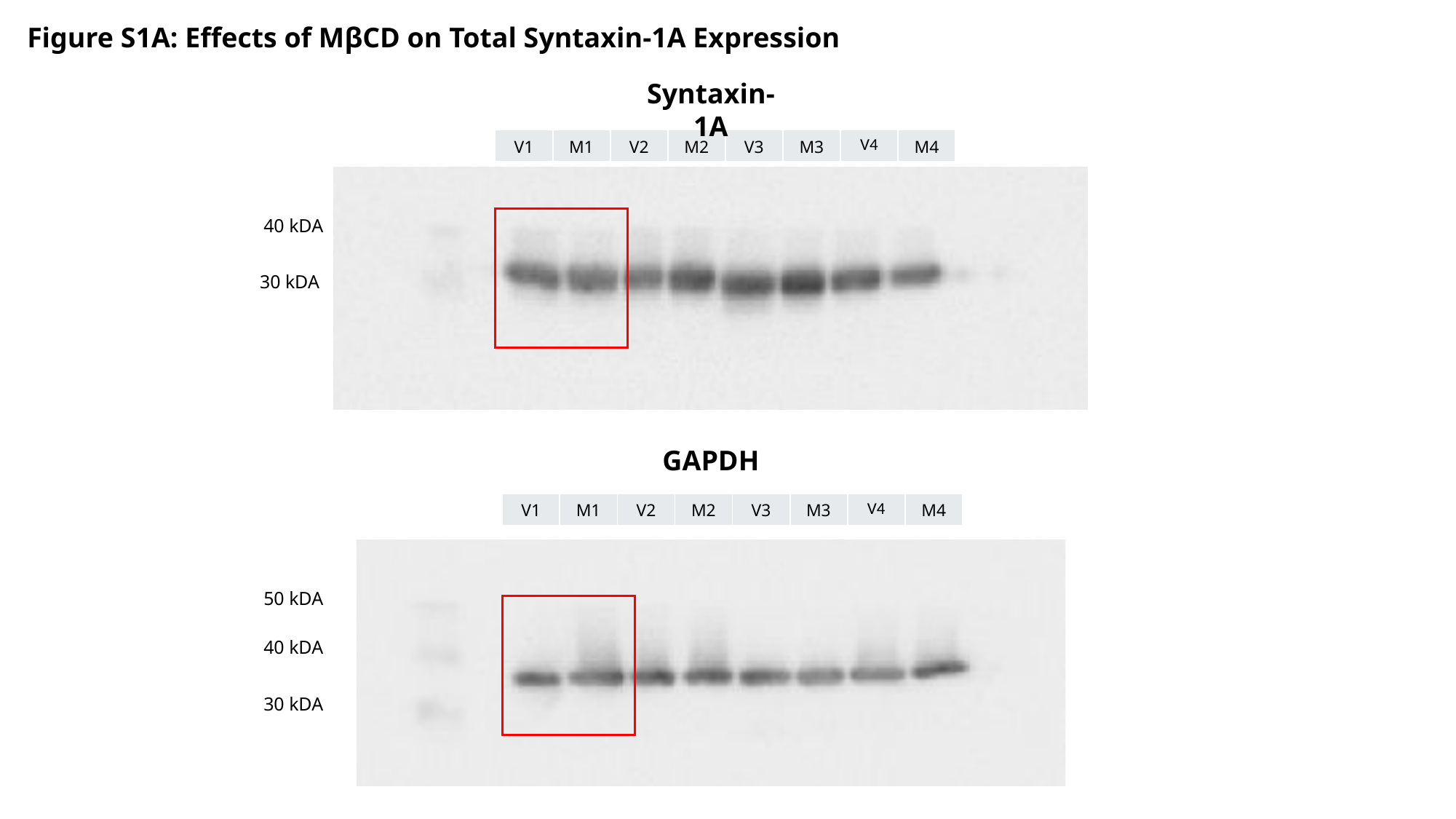

Figure S1A: Effects of MβCD on Total Syntaxin-1A Expression
Syntaxin-1A
| V1 | M1 | V2 | M2 | V3 | M3 | V4 | M4 |
| --- | --- | --- | --- | --- | --- | --- | --- |
40 kDA
30 kDA
GAPDH
| V1 | M1 | V2 | M2 | V3 | M3 | V4 | M4 |
| --- | --- | --- | --- | --- | --- | --- | --- |
50 kDA
40 kDA
30 kDA

## Slide 15
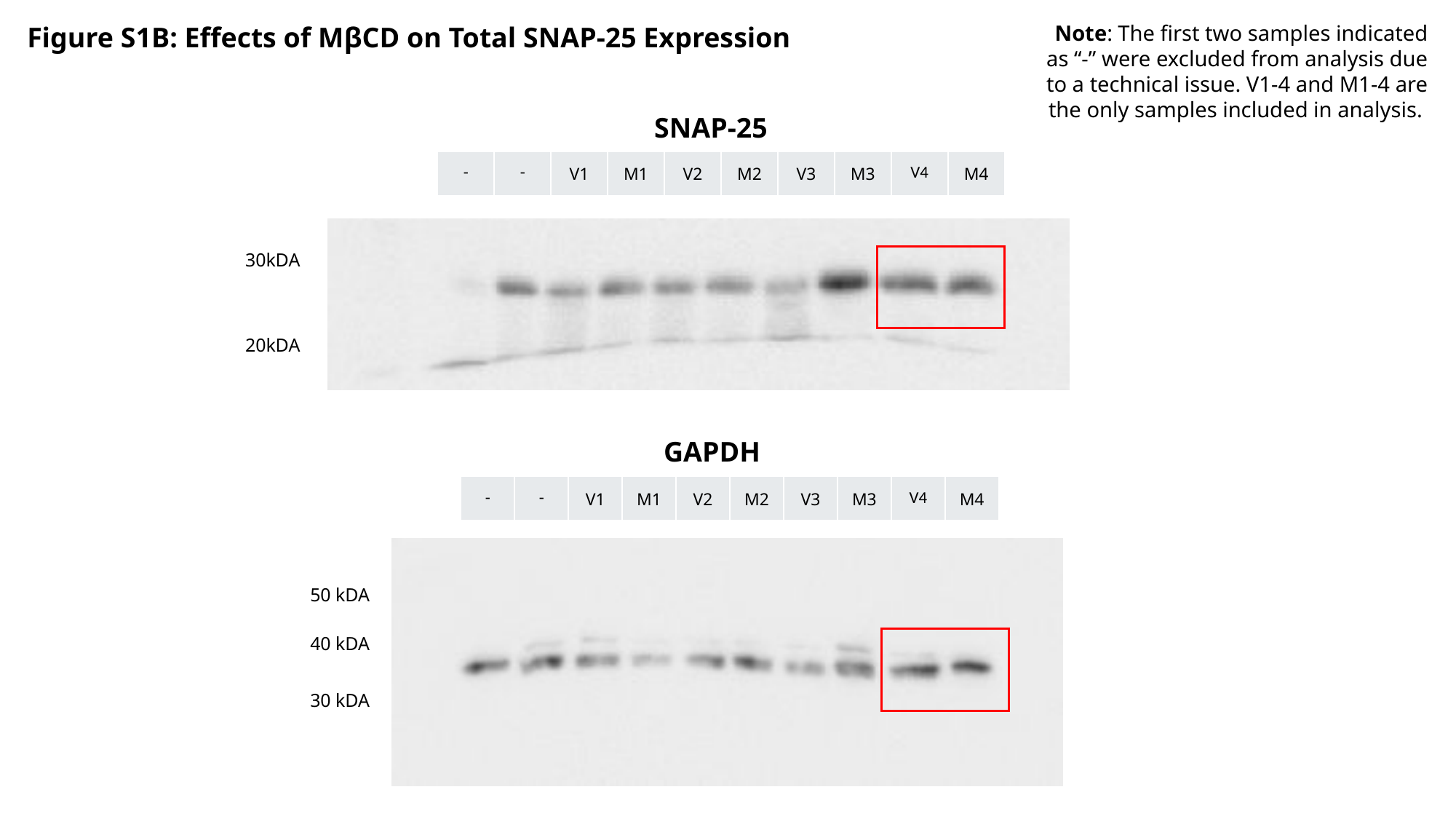

Figure S1B: Effects of MβCD on Total SNAP-25 Expression
Note: The first two samples indicated as “-” were excluded from analysis due to a technical issue. V1-4 and M1-4 are the only samples included in analysis.
SNAP-25
| - | - | V1 | M1 | V2 | M2 | V3 | M3 | V4 | M4 |
| --- | --- | --- | --- | --- | --- | --- | --- | --- | --- |
30kDA
20kDA
GAPDH
| - | - | V1 | M1 | V2 | M2 | V3 | M3 | V4 | M4 |
| --- | --- | --- | --- | --- | --- | --- | --- | --- | --- |
50 kDA
40 kDA
30 kDA

## Slide 16
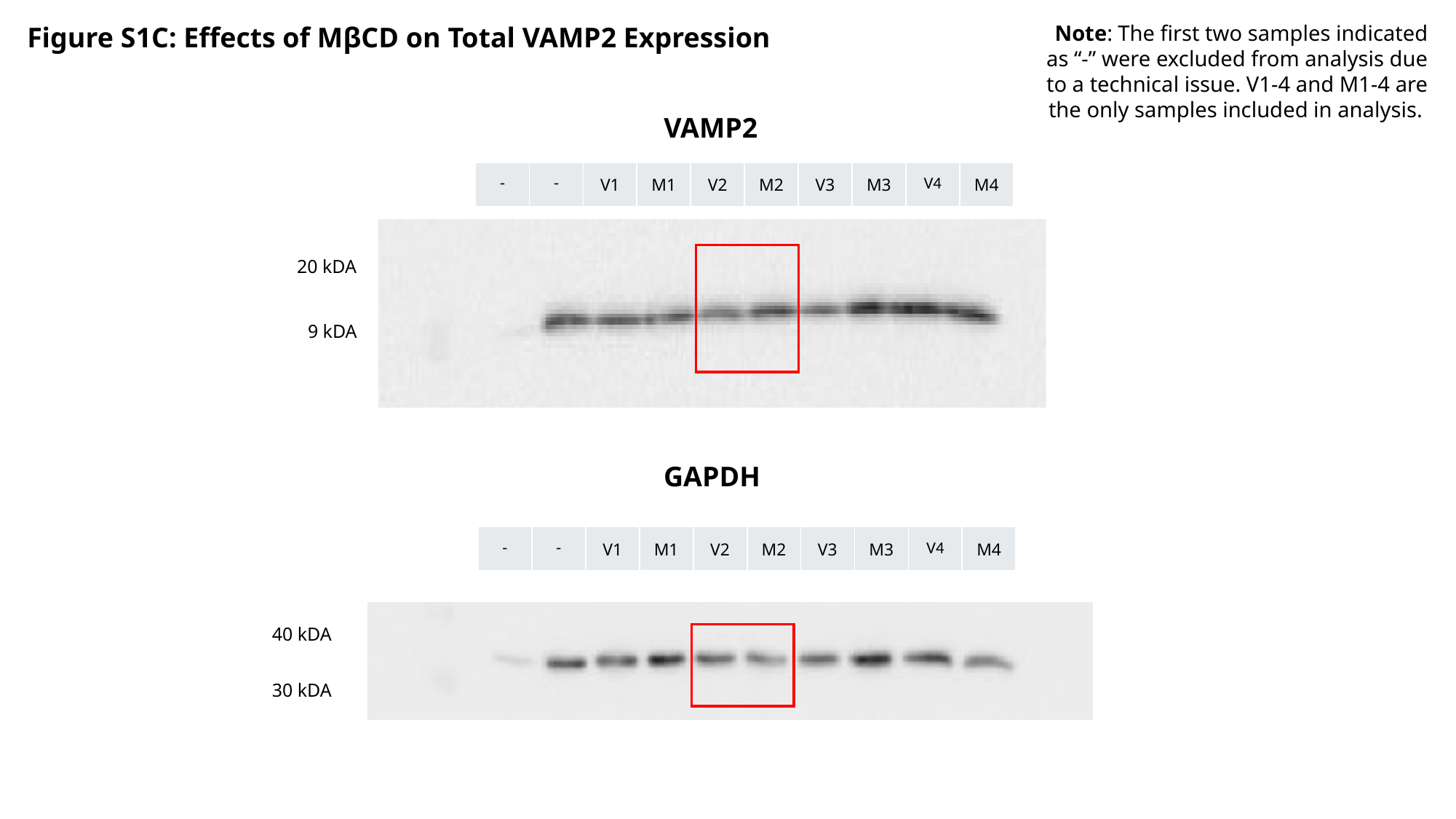

Figure S1C: Effects of MβCD on Total VAMP2 Expression
Note: The first two samples indicated as “-” were excluded from analysis due to a technical issue. V1-4 and M1-4 are the only samples included in analysis.
VAMP2
| - | - | V1 | M1 | V2 | M2 | V3 | M3 | V4 | M4 |
| --- | --- | --- | --- | --- | --- | --- | --- | --- | --- |
20 kDA
9 kDA
GAPDH
| - | - | V1 | M1 | V2 | M2 | V3 | M3 | V4 | M4 |
| --- | --- | --- | --- | --- | --- | --- | --- | --- | --- |
40 kDA
30 kDA

## Slide 17
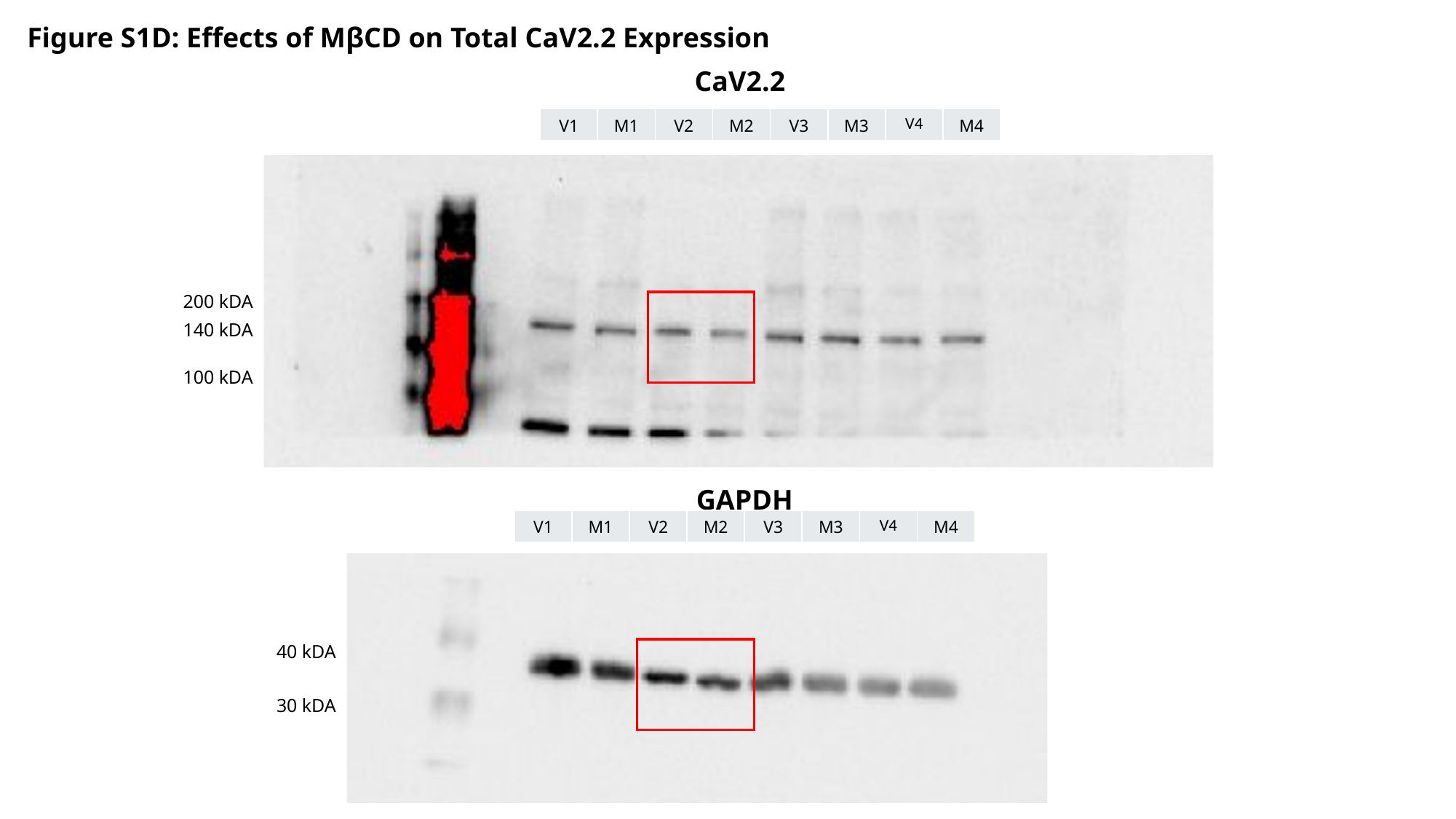

Figure S1D: Effects of MβCD on Total CaV2.2 Expression
CaV2.2
| V1 | M1 | V2 | M2 | V3 | M3 | V4 | M4 |
| --- | --- | --- | --- | --- | --- | --- | --- |
200 kDA
140 kDA
100 kDA
GAPDH
| V1 | M1 | V2 | M2 | V3 | M3 | V4 | M4 |
| --- | --- | --- | --- | --- | --- | --- | --- |
40 kDA
30 kDA

## Slide 18
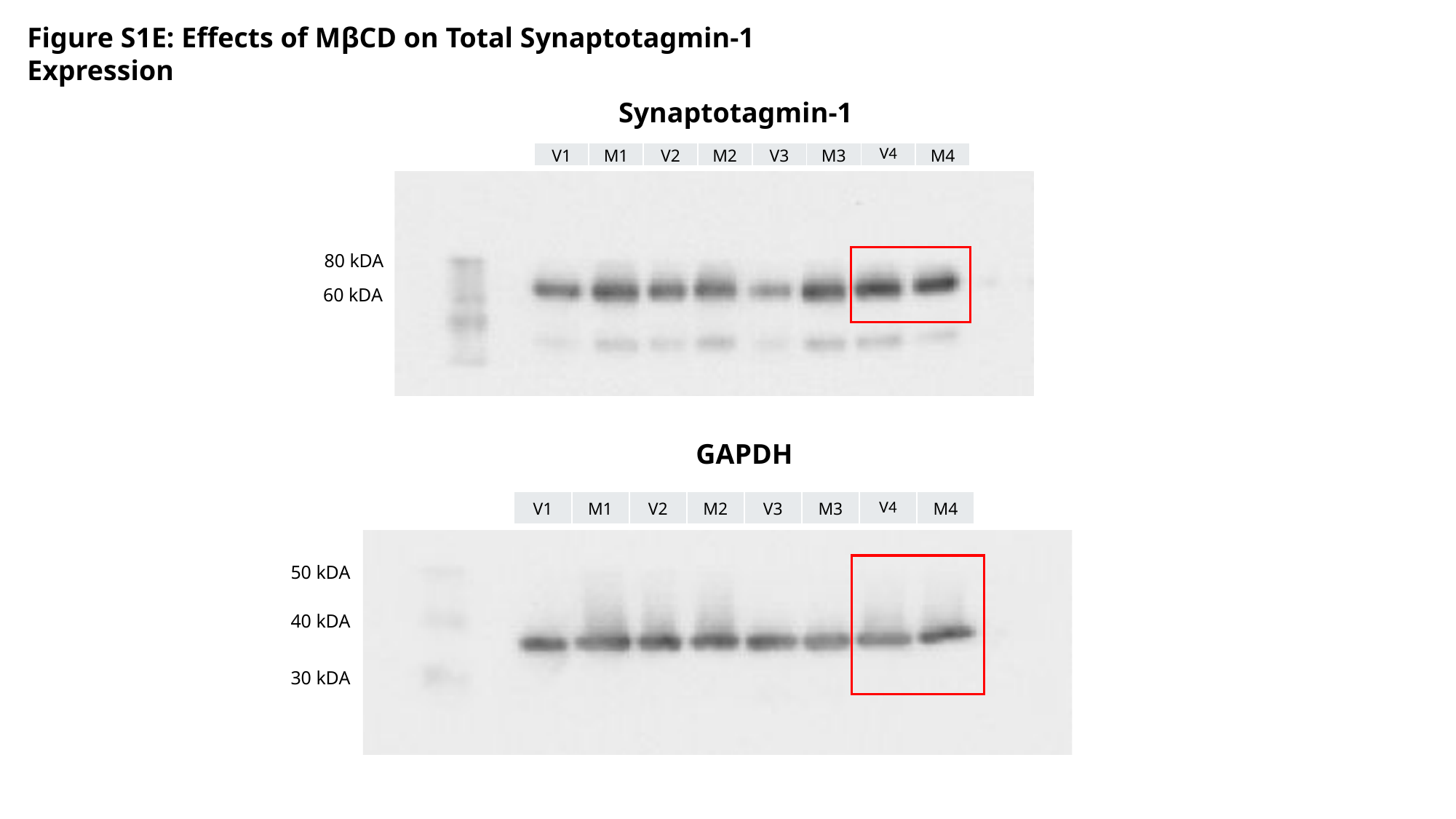

Figure S1E: Effects of MβCD on Total Synaptotagmin-1 Expression
Synaptotagmin-1
| V1 | M1 | V2 | M2 | V3 | M3 | V4 | M4 |
| --- | --- | --- | --- | --- | --- | --- | --- |
80 kDA
60 kDA
GAPDH
| V1 | M1 | V2 | M2 | V3 | M3 | V4 | M4 |
| --- | --- | --- | --- | --- | --- | --- | --- |
50 kDA
40 kDA
30 kDA

## Slide 19
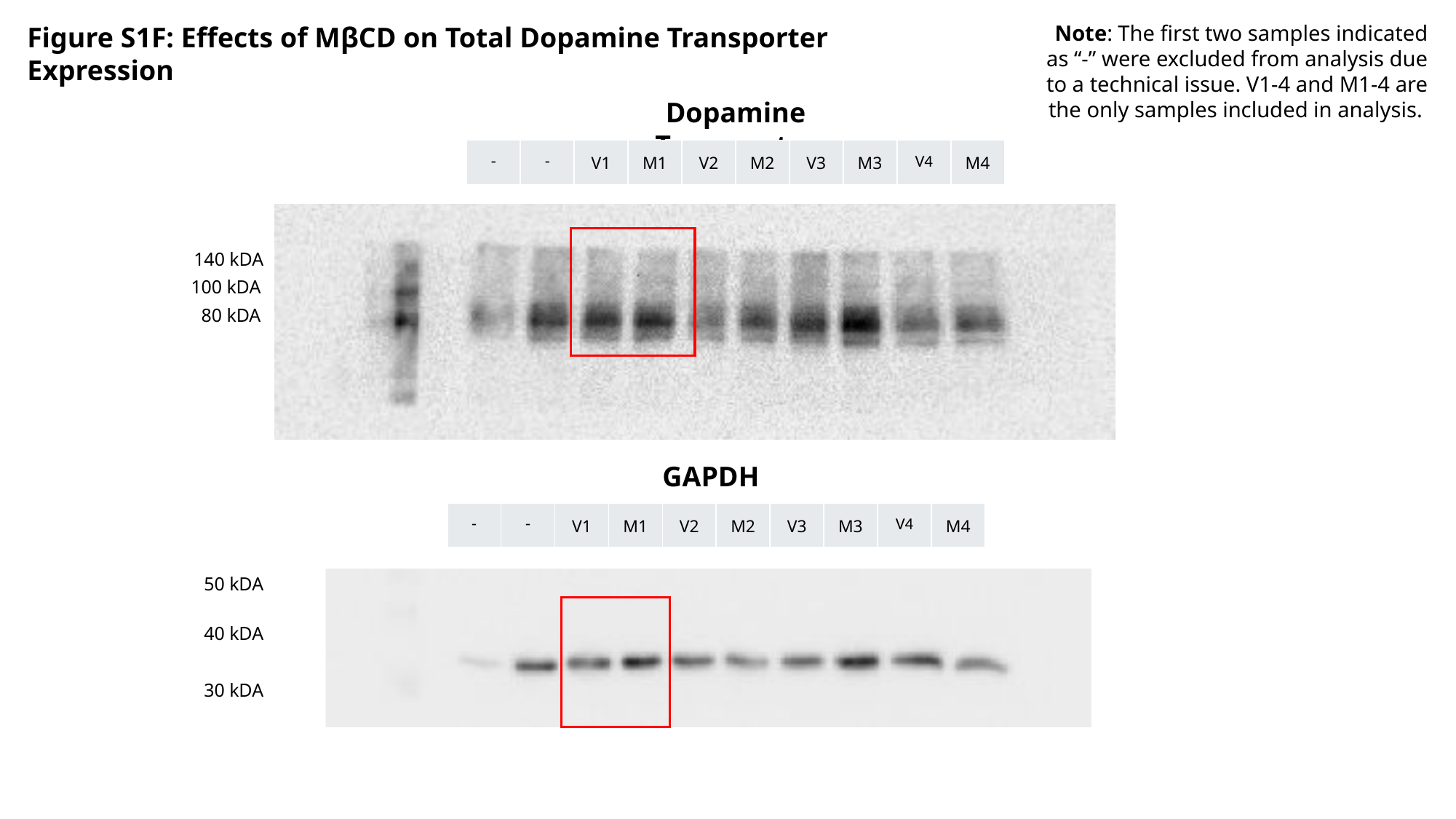

Figure S1F: Effects of MβCD on Total Dopamine Transporter Expression
Note: The first two samples indicated as “-” were excluded from analysis due to a technical issue. V1-4 and M1-4 are the only samples included in analysis.
Dopamine Transporter
| - | - | V1 | M1 | V2 | M2 | V3 | M3 | V4 | M4 |
| --- | --- | --- | --- | --- | --- | --- | --- | --- | --- |
140 kDA
100 kDA
80 kDA
GAPDH
| - | - | V1 | M1 | V2 | M2 | V3 | M3 | V4 | M4 |
| --- | --- | --- | --- | --- | --- | --- | --- | --- | --- |
50 kDA
40 kDA
30 kDA
