# Supplementary material for: Disruption of dopamine transmission by cholesterol depletion is associated with alterations in protein lipid raft partitioning and actin dynamics
Source: Neuropharmacology. Author manuscript; Available in PMC 2026 Jul 1. (PMC13316772; doi:10.1016/j.neuropharm.2026.111078)
Supplement: Supplementary Material 2 [file NIHMS2190795-supplement-Supplementary_Material_2.docx]

**Fig. S1. Loading equal volumes across sucrose density gradient ultracentrifugation fractions produces disproportionately stronger signals in non-raft regions**

To assess protein distribution across sucrose density gradient ultracentrifugation fractions, western blots were initially performed using equal loading volumes across all fractions. However, because non-raft fractions contain significantly more protein than raft fractions, loading equal volumes resulted in disproportionately stronger signal in non-raft fractions, making it difficult to accurately quantify protein abundance across both raft and non-raft regions. To account for these differences in protein content, loading volumes were adjusted across the gradient as follows: 35 μL for raft fractions 4-6 (flotillin-positive), 17.5 μL for intermediate fractions 7-12, and 11.67 μL for non-raft fractions 13-15 (transferrin-positive). For all quantitative analyses, each blot was normalized to its own raft-to-non-raft signal.

**
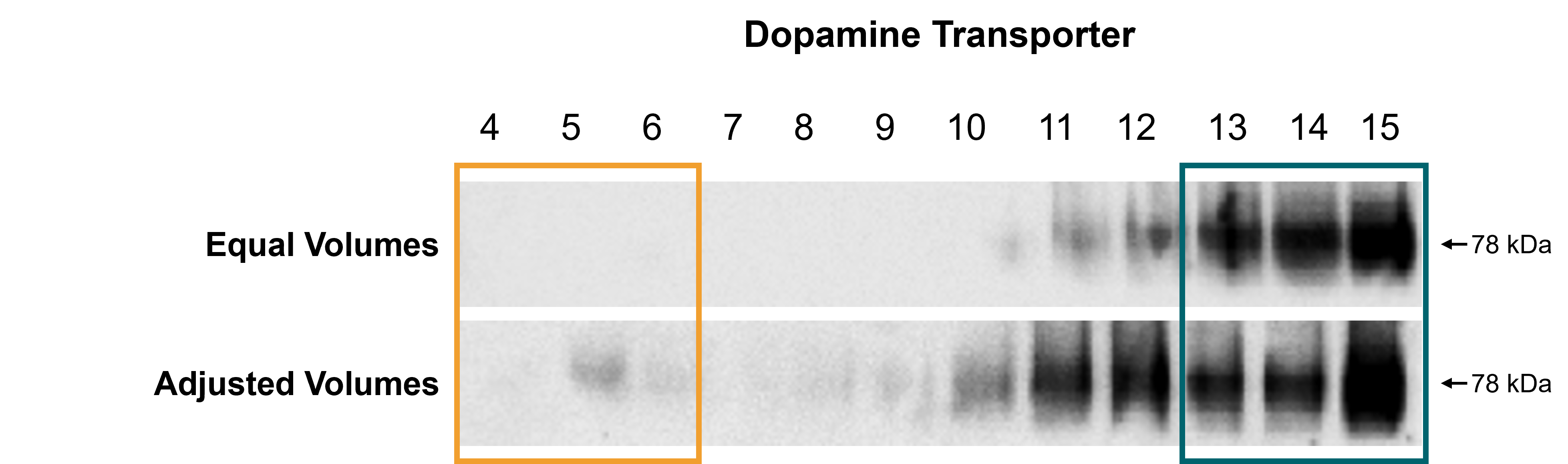
**

**Fig. S1. Equal loading volumes produce disproportionately higher non-raft signals.** Representative western blot showing the distribution of the dopamine transporter (DAT) across raft, intermediate, and non-raft fractions. When equal volumes were loaded across fractions, DAT was not detectable in raft fractions. In contrast, adjusting loading volumes to account for the greater total protein content of non-raft fractions enabled detection of DAT in raft regions.

**Fig. S2. Flotillin is selectively enriched in sucrose density gradient ultracentrifugation fractions 4-6**

We used flotillin as a well-established marker of lipid rafts. To determine the distribution of lipid raft fractions in our sucrose density gradient ultracentrifugation (SDGU) preparation, we immunoblotted flotillin across the first 12 collected fractions. Flotillin was detected exclusively in fractions 4-6. In addition, Figure 2C confirms that flotillin was not detected in fractions 13-15. Based on these findings, fractions 4-6 were designated as raft fractions and fractions 7-15 as non-raft fractions for subsequent SDGU analyses of raft association of proteins.

**
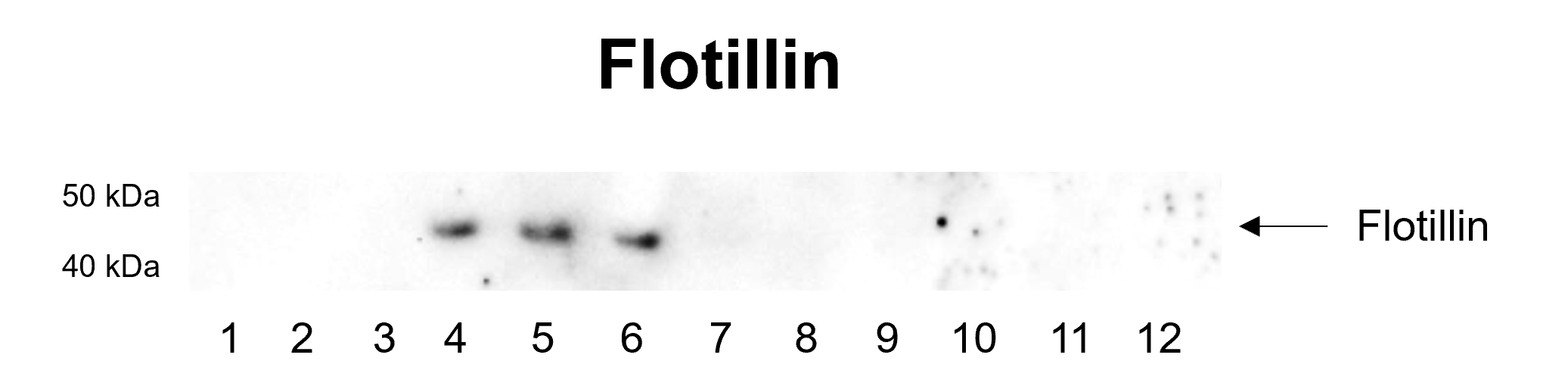
**

**Fig. S2. Flotillin is enriched in SDGU fractions 4-6.** Representative western blot showing the distribution of flotillin across the first 12 fractions collected following SDGU of striatal synaptosomes. Flotillin was detected exclusively in fractions 4-6, identifying these fractions as lipid raft-enriched regions in this preparation.

**Fig. S3. MβCD** **does not alter the total protein expression of proteins involved in dopamine transmission**

Because 3 mM MβCD selectively altered the lipid raft association of several presynaptic proteins, we assessed total protein expression to determine whether these changes reflected altered protein abundance. Immunoblotting for syntaxin‑1A, VAMP2, SNAP‑25, synaptotagmin‑1, CaV2.2, and DAT (normalized to GAPDH; Fig. S3) revealed no significant differences between vehicle- and MβCD-treated samples: syntaxin‑1A, t(5.581) = 0.215, p = 0.837 (Fig. S3A), SNAP‑25, t(3.232) = 1.528, p = 0.218 (Fig. S3B), VAMP2, t(3.892) = 0.960, p = 0.393 (Fig. S3C), synaptotagmin‑1, t(4.056) = 0.817, p = 0.459 (Fig. S3D), Cav2.2, t(6) = 0.228, p = 0.828 (Fig. S3E); and DAT, t(5.472) = 1.424, p = 0.209 (Fig. S3F). These findings indicate that acute cholesterol depletion does not alter the overall abundance of these presynaptic proteins, suggesting that MβCD‑evoked changes in DA release and uptake are not due to reduced protein expression.


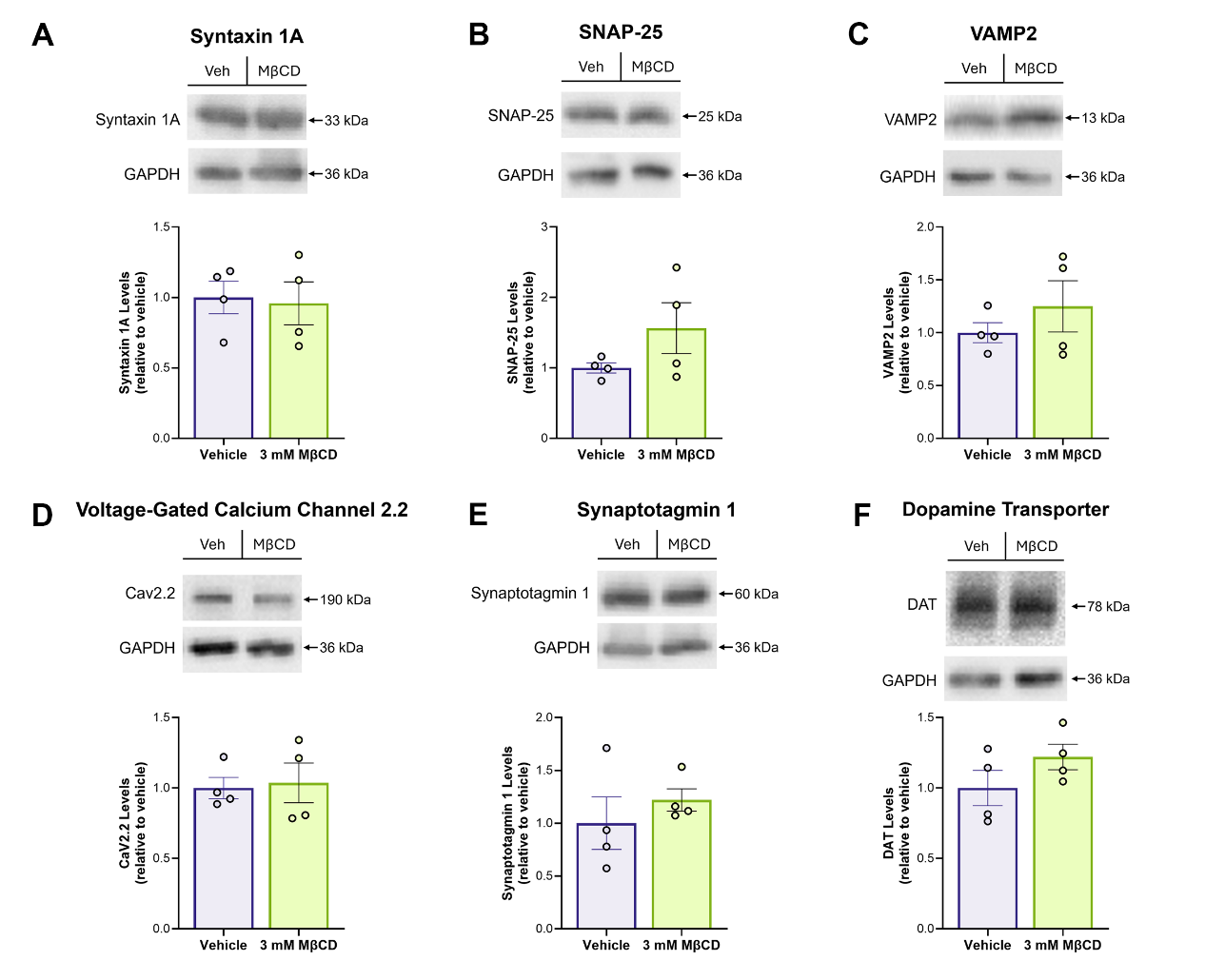


**Fig. S3. MβCD does not alter total protein levels of key presynaptic proteins involved in dopamine release and reuptake.** (A-F) Representative western blots and quantification for syntaxin 1A (A), SNAP-25 (B), VAMP2 (C), CaV2.2 (D), synaptotagmin 1 (E), and DAT (F) following 30 min incubation with 3 mM MβCD or vehicle. Values are normalized to GAPDH and expressed relative to vehicle. Bars show mean ± SEM with points indicating biological replicates (n = 4/group).

**Fig. S4. Actin stabilization with jasplakinolide reduced evoked DA release**

To determine whether stabilization of actin polymerization could be used as a rescue strategy for MβCD-induced reductions in evoked DA release, we tested the effect of jasplakinolide alone on DA transmission using FSCV in slices containing the NAc core. Slices were first allowed to stabilize at baseline, after which jasplakinolide (200 nM) or vehicle (DMSO) was applied for 30 min. Post-treatment signals were normalized to each slice’s pre-treatment signal and expressed as percent of each slice’s baseline. Jasplakinolide significantly reduced evoked DA release compared with control, t(10) = 2.69, p = 0.022 (Fig. S4).


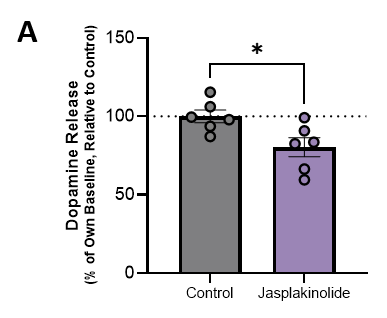


**Fig. S4. Jasplakinolide reduced evoked DA release in rat NAc slices.** Evoked DA release was measured by FSCV following baseline stabilization and subsequent treatment with control (DMSO; N = 4) or jasplakinolide (200 nM; N = 4) for 30 min. Data are expressed as percent of each slice’s own baseline, relative to control. Jasplakinolide significantly reduced evoked DA release compared with control. *p ≤ 0.05. N = number of animals; 1-2 slices/animal. Data are presented as mean ± SEM.
